# PGRMC1-dependent lipophagy promotes ferroptosis in paclitaxel-tolerant persister cancer cells

**DOI:** 10.1186/s13046-021-02168-2

**Published:** 2021-11-08

**Authors:** Ji Hyeon You, Jaewang Lee, Jong-Lyel Roh

**Affiliations:** Department of Otorhinolaryngology-Head and Neck Surgery, CHA Bundang Medical Center, CHA University School of Medicine, Seongnam, Gyeonggi-do 13496 Republic of Korea

**Keywords:** Progesterone receptor membrane component 1, Ferroptosis, Fatty acid oxidation, Lipophagy, Tubulin detyrosination

## Abstract

**Background:**

Progesterone receptor membrane component 1 (PGRMC1) is a heme-binding protein inducing dimerization with cytochrome P450, which mediates chemoresistance. Increased PGRMC1 expression is found in multiple types of resistant cancers, but the role of PGRMC1 in the ferroptosis of cancer cells remains unrevealed. Therefore, we examined the role of PGRMC1 in promoting ferroptosis in paclitaxel-tolerant persister cancer cells (PCC).

**Methods:**

The effects of ferroptosis inducers and PGRMC1 gene silencing/overexpression were tested on head and neck cancer (HNC) cell lines and mouse tumor xenograft models. The results were analyzed about cell viability, death, lipid ROS and iron production, mRNA/protein expression and interaction, and lipid assays.

**Results:**

PCC had more free fatty acids, lipid droplets, and fatty acid oxidation (FAO) than their parental cells. PCC was highly sensitive to inhibitors of system xc^−^ cystine/glutamate antiporter (xCT), such as erastin, sulfasalazine, and cyst(e)ine deprivation, but less sensitive to (1S,3R)-RSL3. PGRMC1 silencing in PCC reduced ferroptosis sensitivity by xCT inhibitors, and PGRMC1 overexpression in parental cells increased ferroptosis by xCT inhibitors. Lipid droplets were degraded along with autophagy induction and autophagosome formation by erastin treatment in PCC. Lipophagy was accompanied by increased tubulin detyrosination, which was increased by SIRT1 activation but decreased by SIRT1 inhibition. FAO and lipophagy were also promoted by the interaction between lipid droplets and mitochondria.

**Conclusion:**

PGRMC1 expression increased FAO and ferroptosis sensitivity from in vivo mice experiments. Our data suggest that PGRMC1 promotes ferroptosis by xCT inhibition in PCC.

**Supplementary Information:**

The online version contains supplementary material available at 10.1186/s13046-021-02168-2.

## Condensed abstract

Paclitaxel-tolerant persister cancer cells (PCC) had PGRMC1 upregulation related to increased free fatty acids, lipid droplets, and fatty acid oxidation. PGRMC1 expression substantially increased ferroptosis by xCT inhibition via lipophagy and tubulin detyrosination, whereas PGRMC1 silencing decreased ferroptosis: this suggests that PGRMC1 expression promotes ferroptosis in PCC.

**Supplementary Information:**

The online version contains supplementary material available at 10.1186/s13046-021-02168-2.

## Background

Despite the development of various anti-cancer agents, cancer cells can evade effective treatment via some of the ways from pre-existing resistance, de novo mutations, and drug-tolerant persisters [1]. Drug-tolerant persister cancer cells are small surviving fractions evading from chemotherapeutic drugs that cause a significant drawback of conventional or targeted therapy. A slow glowing persister cancer cells are more resistant to anti-cancer drugs [2]. In response to treatment toxicity, tumor cell plasticity is attended with the donning molecular disguise of genetic, transcriptional, chromatin, epigenetic, and microenvironmental reprogramming [3]. Understanding the resistant mechanisms can lead to incremental innovation for the development of effective anti-cancer strategies. Drug-tolerant persister cancer cells have characteristics of a high mesenchymal cell state that lead to dependency on a lipid peroxidase pathway involving glutathione peroxidase 4 (GPX4) [4]. Acquired drug resistance can be overcome by ferroptosis inducers targeting xCT or GPX4, the central regulator of ferroptosis [5, 6].

The lipid peroxidase pathway utilizes glutathione (GSH) which is a seminal cellular antioxidant for eliminating lipid reactive oxygen species (ROS) [7]. GSH is generated from cysteine transported by system xc^−^ cystine/glutamate antiporter (xCT) [7]. Polyunsaturated fatty acids (PUFAs) are essential sources to produce lipid peroxidation by multiple inner bonds of ROS [8]. Consequently, excessive lipid peroxidation disturbs cellular membrane integrity and causes cell death [9]. Ferrous iron catalyzes the Fenton reaction as an electron donor, leading to a new form of necrotic cell death, ferroptosis [10].

Progesterone receptor membrane component 1 (PGRMC1) is a heme-binding protein that binds and modulates the activity of cytochrome P450 enzymes, which may impact multiple biochemical pathways and drug metabolism [11]. PGRMC1 involves the diverse functions of steroidogenesis, progesterone (P4) signaling, membrane trafficking, and mitotic spindle and cell cycle regulation [12]. Increased PGRMC1 expression is found in various tumors, including lung, colon, thyroid, breast, ovary, and cervix [13]. Besides, PGRMC1 induces autophagy via binding to microtubule-associated proteins 1 light chain 3 (LC3), an essential component for the degradative activity of autophagy [14]. PGRMC1 expression influences altered lipid and glycolytic metabolism, resistances to apoptosis by chemotherapeutic agents, epithelial-mesenchymal transition (EMT), invasion, and metastasis [12]. PGRMC1 is also involved in hormone and cholesterol synthesis and drug metabolism, which mediates chemoresistance [15]. The role of PGRMC1 related to autophagy and lipid alteration might be liked to ferroptosis, an autophagic process based on the iron-dependent accumulation of excessive lipid peroxidation [10].

Increased PGRMC1 expression is found in multiple types of resistant cancers, but the role of PGRMC1 in cancer cell ferroptosis remains unrevealed. We developed drug-tolerant persister cancer cells from parental head and neck cancer (HNC) cells. The persister cells exhibited a highly increased expression of PGRMC1 that might contribute to the acquisition of drug resistance but became vulnerable to ferroptosis by xCT inhibitors. Therefore, we examined the role of PGRMC1 in promoting ferroptosis in paclitaxel-tolerant persister cancer cells (PCC).

## Methods

### Cell culture and drug-tolerant persister cancer cell derivation

HNC cell lines, namely AMC HN3 and HN4, were used for our experiments [16]. The cell lines were authenticated by short tandem repeat-based DNA fingerprinting and multiplex polymerase chain reaction (PCR). The cells were cultured in Eagle’s minimum essential medium (Thermo Fisher Scientific, Waltham, MA, USA) supplemented with 10% fetal bovine serum, penicillin, and streptomycin at 37 °C in a humidified atmosphere containing 5% CO_2_. Drug-tolerant persister cancer cells were derived from HN3 and HN4 cells with 10 nM paclitaxel (Sigma-Aldrich, St. Louis, MO, USA). Paclitaxel was treated for 6 days with a new drug added every 3 days, and this was repeated in regrown cells after no treatment for 38 days. Re-derived persister and parental cells were used in experiments. PCC was selected by 10 nM paclitaxel every 2 weeks to maintain drug tolerance characteristics.

### Cell viability and death assays

Cell viability was measured in the cells that were subjected to (1S,3R)-RSL3) (19,288; Cayman Chemical Co., Ann Arbor, MI, USA), erastin (S7242; Selleckchem, Houston, TX, USA), sulfasalazine (S0883; Sigma-Aldrich), or an equivalent amount of dimethyl sulfoxide (DMSO), or were cultured in the conditioned media with no cysteine and cystine (cyst(e)ine, 1,641,454; MP Biomedicals, Irvine, CA, USA). After exposure, cell viability was assessed using cell counting kit-8 (CCK-8) (CK04; Dojindo Molecular Technologies, Inc., Tokyo, Japan) according to the manufacturer’s protocol. The cells were incubated with the CCK-8 solution for 1 h, and the cell viability was measured at the absorbance of 450 nm using a SpectraMax M2 microplate reader (Molecular Devices, Sunnyvale, CA, USA).

After exposure to the agents, cell death was assessed via SYTOX Green (S34860; Thermo Fisher Scientific) staining. The samples were washed three times with Hanks’ balanced salt solution without calcium and magnesium (HBSS, 14025076; Thermo Fisher Scientific), then staining cells in each plate with 5 μM SYTOX Green in HBSS for 20 min. The stained cells were observed using a ZEISS fluorescent microscope (Axiovert 200 M; Oberkochen, Germany) and analyzed using ImageJ software (NIH, Bethesda, MD, USA). The mean SYTOX Green-positive fractions were compared with those of the control group.

### Measurement of lipid and mitochondrial reactive oxygen species

Lipid reactive oxygen species (ROS) generation was measured by adding 5 μM BODIPY™ 581/591 C11 (a lipid peroxidation sensor, D3861; Thermo Fisher Scientific) for 30 min 37 °C. The ROS levels were analyzed using a CytoFLEX flow cytometer (Beckman Coulter, Brea, CA, USA), (non)oxidized and oxidized forms were confirmed by ZEISS fluorescent microscope. Image quantification was performed using ImageJ software. For mitochondrial ROS, cells were seeded in 60 mm dishes. After indicated drug treatment, cells were incubated with 5 μM mitoSOX™ Red (M36008; Thermo Fisher Scientific) for 20 min. MitoSOX™ Red was measured by ZEISS fluorescent microscope. The quantification of fluorescence intensity was performed using ImageJ software.

### Glycolysis and glutamate assays

Glycolysis assay was measured using a glycolysis assay kit (ab197244; Abcam) at 380 nm excitation and 615 nm emission using a SpectraMax M2 microplate reader. The glycolytic effect was calculated through extracellular acidification (ECAR) using a microplate fluorometer at 15 min intervals and was examined from ECAR assay at 120 min. All examinations were operated on in 5 × 10^5^ cells per sample following the manufacturer’s protocol. Glutamate contents were measured using a glutamate assay kit (ab83389; Abcam) following the manufacture’s protocol.

### Measurement of GSH synthesis and intracellular iron

Intracellular GSH levels in HNC cell lysates were measured using a GSH/GSSG assay kit (EGTT-100; BioAssay Systems, Hayward, CA, USA) according to the manufacturer’s instructions.

Labile iron pool (LIP) assay was measured by using calcein acetoxymethyl ester (354,217; Corning Inc., Corning, NY, USA) and iron chelator, deferoxamine (ab120727; Abcam, Cambridge, UK). The cells were loaded with calcein-AM (8 μg/ml) for 30 min at 37 °C and then washed with HBSS. Deferoxamine was added at a final concentration of 100 μM to remove iron from calcein, causing dequenching. The change in fluorescence following the addition of deferoxamine was used as an indirect measure of the LIP. Fluorescence was measured at 485 nm excitation and 535 nm emission with a VICTOR X3 microplate reader (PerkinElmer, Waltham, MA, USA) and ZEISS fluorescent microscope.

### Measurements of autophagic flux

The cells were seeded and were treated with erastin or other agents with or without 30 nM Wortmannin (W1628; Sigma-Aldrich). All cells were stained with LysotrackerTM Red DND-99 (L7528; Thermo Fisher Scientific) to assess the later process of autophagy. Co-localization of LC3-GFP-puncta and lysosome was confirmed using the ZEISS LSM 880 confocal microscope. Also, autophagy-related molecules were confirmed by immunoblotting.

### Measurements of free fatty acid

For free fatty acid (FFA) quantification, parental HNC cells and PCC were seeded in 100 mm dishes. Then, cells were treated with or without ferroptosis inducers. Intracellular FFA was measured using PicoSens™ Free Fatty Acid Quantification Kit (BM-FFA-100; BIOMAX, Seoul, Republic of Korea) according to the manufacturer’s instructions.

### GC/MS analysis

PCC and parental cells were seeded in culture medium in 150-mm tissue culture dishes and were harvested in 2 days with a rubber-tipped cell scraper. The cells were washed, centrifuged twice with 1× phosphate-buffered saline, and transferred to tubes in an equal number of 5 × 10^6^ cells. The cells proceeded with lipid extraction after flash-freezing in LN2. For fatty acid methyl esters (FAME) analysis, cells were lyophilized and ground into fine powders. Fatty acids were extracted using 2 mL methylation mixture (MeOH:Benzene:DMP (2,2-Dimethoxy-propane):H_2_SO_4_ = 39:20:5:2) and 1 mL heptane, 80 °C for 2 h. Then, supernatants were analyzed using GC/MS (Agilent 7890 GC System; Agilent Technologies, Santa Clara, CA, USA). The analysis condition was as follows: column (DB-23, 120 mm*0.25 mm* 0.25 μm; Agilent), injector (250 °C), detector (FID-280 °C, H_2_ 35 ml/min, air 350 ml/min, He 10 ml/min), STD (CRM47885; Supelco 37 component FAME Mix; Supelco, Inc., Bellefonte, PA, USA), and ISTD (P6125; Pentadecanoic acid) (Sigma-Aldrich) [17]. After GC/MS analysis, all samples were normalized.

### RNA interference and gene transfection

HNC cells were seeded for gene silencing or overexpression. Cells were transfected 24 h later with 10 nmol/L small-interfering RNA (siRNA) targeting human SIRT1 or scrambled control siRNA (Integrated DNA Technologies, Coralville, IA, USA) using Lipofectamine RNAiMAX reagent (13,778,075; Thermo Fisher Scientific). PCC was stably transduced with short hairpin RNA (shRNA) targeting PGRMC1 (pGPU6/Neo, GenePharma, Shanghai, China) using Lipofectamine 3000 reagent (L3000001; Thermo Fisher Scientific). HN3 and HN4 cells were seeded and stably transfected with a control pcDNA3.1 plasmid (V790–20; Addgene, Watertown, MA, USA) or pcDNA3.1-PGRMC1 plasmid by using Lipofectamine 3000 reagent. The levels of PGRMC1 and SIRT1 expression were confirmed via western blotting. pEGFP-LC3 (21,073; Addgene) was stably transfected into HN4 parental cells, HN4-PGRMC1 plasmid, HN4PCC, and HN4PCC-shPGRMC1 using Lipofectamine 3000 reagent.

### Reverse transcription-quantitative PCR and methylation-specific PCR

HNC cells were cultured with 70% confluence in 6-cm tissue culture dishes. Total RNA from HNC cells was isolated using a total RNA extraction kit (K-3140; Bioneer, Daejeon, Republic of Korea) according to the manufacturer’s instructions. A reverse transcription-quantitative polymerase chain reaction (RT-qPCR) was performed using a SensiFAST™ SYBR® No-ROX Kit (BIO-98050; Bioline International, Toronto, Canada) after cDNA synthesis with a SensiFAST™ cDNA Synthesis Kit (BIO-65054; Bioline International). PGRMC1, SIRT1, and ACTB were amplified, and the relative target mRNA levels were determined using mathematical expression 2^−(ΔΔCt)^. All data were normalized against ACTB mRNA levels. Real-time PCR was performed with ViiA™ 7 Real-Time PCR System (Applied Biosystems, Foster City, CA, USA). Methylation-specific PCR (MSP) indicated methylated promoter level in bisulfite-treated genomic DNA. Genomic DNA from HNC cells was extracted by a genomic DNA extraction kit (YGB100; Real Biotech Co., Taipei, Taiwan). Extracted genomic DNA was converted into a bisulfite form using a BisulFlash DNA Modification Kit (P-1026-050; EpiGentek, Farmingdale, NY, USA). The degree of methylation was determined in SIRT1 by RT-qPCR using a Methylamp MS-qPCR Fast Kit (P-1028-100; EpiGentek).

### Immunoblotting and immunostaining

Cells were plated and grown with 70% confluence and then treated with indicated drugs or not. Cells were lysed at 4 °C in a cell lysis buffer (9803; Cell Signaling Technology, Danvers, MA, USA) with a protease/phosphatase inhibitor cocktail (5872; Cell Signaling Technology). A total of 10–40 μg protein was resolved by SDS-PAGE on 10–15% gels; the resolved proteins were then transferred to nitrocellulose or polyvinylidene difluoride membranes and probed with primary and secondary antibodies. The following primary antibodies were used: PGRMC1 (K004086P; Solarbio Life Science, Beijing, China), CD36 (K004214P; Solarbio), ATGL (K004384P; Solarbio), PLIN2 (K004402P; Solarbio), ACC (3662; Cell Signaling Technology Co., Danvers, MA, USA), FASN (K001685P; Solarbio), CPT1A (K000391P; Solarbio), AMPK (2532; Cell Signaling), pAMPK (2531; Cell Signaling), 4-HNE (MA5–27570; Invitrogen), LC3B (K002189P; Solarbio), ATG5 (K106671P; Solarbio), p62 (K005444P; Solarbio), SIRT1 (sc74465; Santa Cruz Biotechnology, Santa Cruz, CA, USA), TTL (K009740P; Solarbio), TCP1 (K003097P; Solarbio), tyrosinated α-tubulin (ABT171; Merck Millipore, Burlington, MA, USA), detyrosinated α-tubulin (AB3201; Merck Millipore), xCT (K009230P; Solarbio), GPX4 (K006597P; Solarbio), ACSL4 (K004812P; Solarbio), and β-actin (BS6007M; BioWorld, Atlanta, GA, USA). β-actin served as the total loading control.

The cells were also immunostained with an antibody against PGRMC1 (1:200; Solarbio) or detyrosinated α-tubulin (1:200; Merck Millipore). The cells were co-stained with BODIPY™ 493/503 for lipid droplets (D3922; Thermo Fisher Scientific), LysoTracker™ Deep Red (L12492; Thermo Fisher Scientific), or MitoTracker™ (M7510; Thermo Fisher Scientific). Nuclei were blue-stained with 4′,6-diamidino-2-phenylindole (DAPI).

### Tumor xenograft

All animal study procedures were performed by protocols approved by the Institutional Animal Care and Use Committee (IACUC). Six-week-old athymic BALB/c male nude mice (nu/nu) were purchased from OrientBio (Seoul, Republic of Korea). HN4 cells with transfection of PGRMC1 overexpression or control vector and HN4PCC with shPGRMC1 or control vector were subcutaneously injected into the bilateral flank of nude mice. When gross nodules were detected in tumor implants, mice were subjected to different treatments: vehicle or sulfasalazine (250 mg/kg daily per intraperitoneal route) [18]. Erastin was not used due to its physiological instability, and instead, sulfasalazine was used in our in vivo experiment [4]. Each group included six mice. Each mouse’s tumor size and weight were measured twice a week, and tumor volume was calculated as (length × width^2^)/2. After mice were sacrificed, tumors were isolated and analyzed by staining lipid droplets. The values were compared among differently treated tumors.

### The Cancer Genome Atlas (TCGA) dataset and statistical analysis

The expression levels of PGRMC1 mRNA were obtained from the normal mucosa (*n* = 44) and HNC (*n* = 499) datasets of TCGA. The tumor and survival data were analyzed to find the correlation between the expression level of PGRMC1 mRNA and their survival outcomes.

Data were presented as mean ± standard deviation (s.d.). The statistically significant differences between the treatment groups were assessed using Mann–Whitney *U*-test or analysis of variance (ANOVA) with the Bonferroni post-hoc test. The median values of low and high expression levels of PCBP1 mRNA were determined and compared using a *t*-test. The cutoff value of PGRMC1 was decided at the lowest *P* values for overall survival. Univariate Cox proportional hazards regression analyses were used to identify associations between PGRMC1 mRNA expression levels and overall survival in the HNC cohort. The Kaplan–Meier and log-rank tests were used to determine and statistically compare the survival rates, respectively. All statistical tests were two-sided, and a *P*-value of < 0.05 was considered to be statistically significant. The statistical tests were performed using IBM SPSS Statistics version 22.0 (IBM, Armonk, NY, USA).

## Results

### PCC has a metabolic shift to fatty acid oxidation

**Fig. 1:**
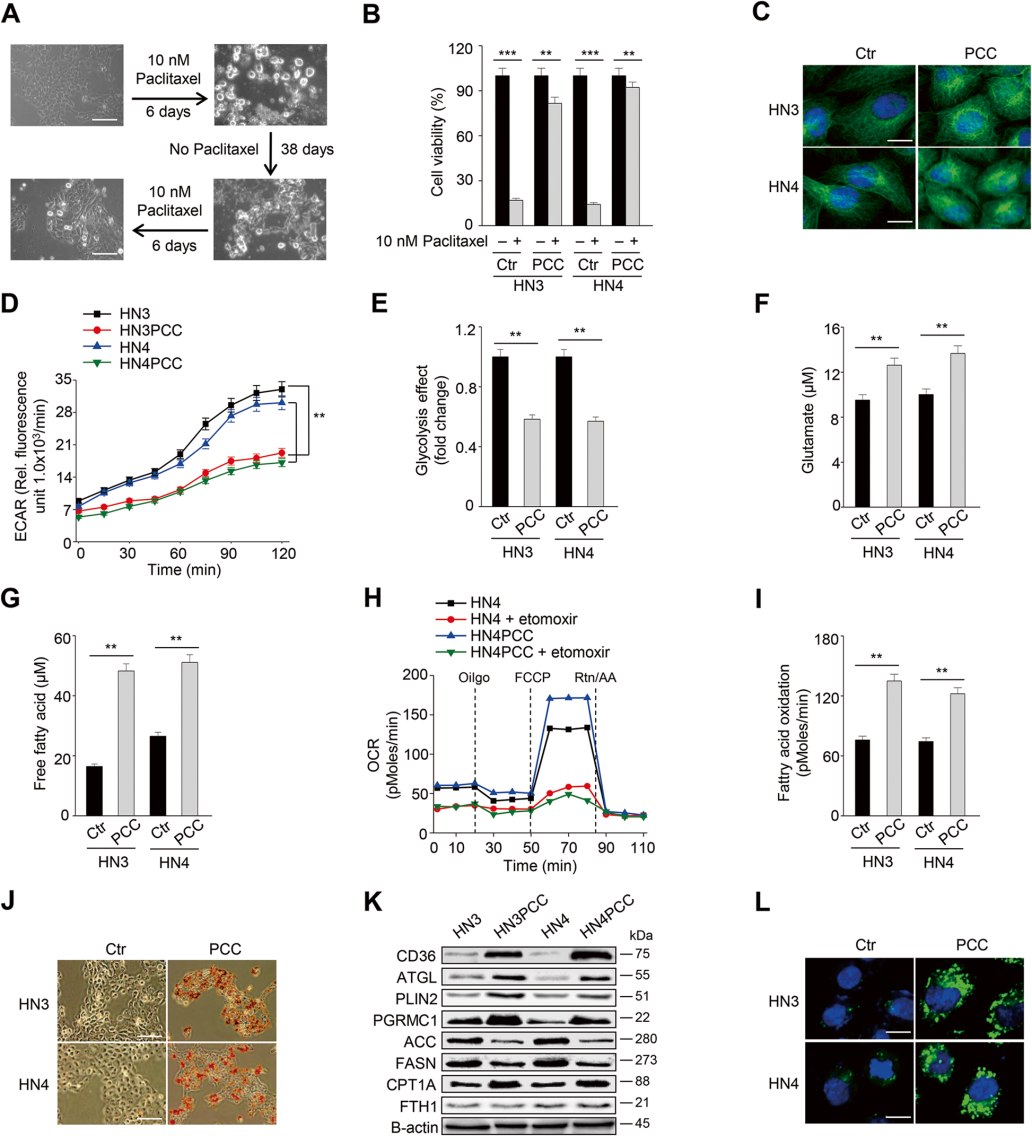
Paclitaxel-tolerant persister cancer cells (PCC) have a metabolic shift to fatty acid oxidation. **A** PCC was made from HN3 and HN4 cells using 10 nM paclitaxel for 6 days and then was maintained without paclitaxel for 38 days before the second 6-day drug treatment to get re-derived PCC. Scale bar 10 μm. **B** Cell viability was measured using cell counting kit-8 (CCK-8) assay after 10 nM paclitaxel treatment for 48 h in parental cells (ctr) and PCC. Data are means and s.d. from three technical replicates. **P < 0.01, ***P < 0.001 relative to DMSO control. **C** Immunostaining of α-tubulin (green) in HN3 and HN4 parental cells and PCC. Nuclei (blue) were stained with 4′,6-diamidino-2-phenylindole (DAPI). Scale bar 10 μm. **D** and **E** Extracellular acidification rate (ECAR) assay in HN3 and HN4 parental cells and PCC. ECAR was measured using a microplate fluorometer at 15 min intervals, and the glycolysis effect was examined from ECAR assay at 120 min. **P < 0.01 relative to parental cells. **F** Cellular glutamate was quantified in parental cells and PCC. **P < 0.01 relative to parental cells. **G**-**I** Free fatty acids and fatty acid oxidation (FAO) were measured in parental cells and PCC. FAO was quantified via assessing changes in oxygen consumption (OCR) and calculated as a formula (sample untreated with etomoxir minus sample treated with 10 μM etomoxir). Data are means and s.d. and from three technical replicates. **P < 0.01 relative to parental cells. **J**-**L** Oil red O staining, immunoblotting, and lipid droplet staining in parental cells and PCC. Scale bars 100 μm (**J**) and 10 μm (**L**)

Drug-tolerant persister cancer cells were developed from the treatment of paclitaxel, a first-line taxane anti-cancer agent for various cancer types in the head and neck, esophagus, lung, ovary, cervix, breast, pancreas, and others [19]. Two HNC cell lines, HN3 and HN4, were treated with a cytotoxic dose (10 nM) of paclitaxel for 6 days, after which remained only a tiny population of surviving persister cancer cells. The cells were regrown without drugs for 38 days and then were re-treated with 10 nM paclitaxel for 6 days to acquire drug-tolerant persister traits (Fig. 1A). Cell viability significantly increased, and cell death significantly decreased in HN3 and HN4 PCC when compared with those of HN3 and HN4 parental cells (*P* < 0.01) (Figs. 1B and S1A).

PCC had different properties from their parental HNC cells. Microtubules were disoriented in PCC but not in parental cells (Fig. 1C). ECAR and glycolysis effect of PCC were significantly lower than those of parental cells, whereas glutamate contents of PCC were markedly higher than those of parental cells (*P* < 0.01) (Fig. 1D–F). Free fatty acids and fatty acid oxidation (FAO) significantly increased in PCC compared to parental cells (*P* < 0.01) (Fig. 1G–I). Lipids were more deposited, and lipid droplets substantially increased in PCC than parental cells (Fig. 1J and L). Also, intracellular labile iron pool increased modestly in PCC than parental cells (*P* < 0.05) (Fig. S1B). On immunoblotting, molecules related to fatty acids uptake and FAO, such as CD36, ATGL, PLIN2, PGRMC1, and CPT1A, increased (Fig. 1K). Still, molecules related to fatty acid synthesis (FAS), such as ACC and FASN, decreased in PCC compared to parental cells. FTH1 was not significantly changed. Taken together, PCC showed more metabolic trend shifting to FAO than their parental cells.

### PCC is vulnerable to xCT inhibitors

**Fig. 2:**
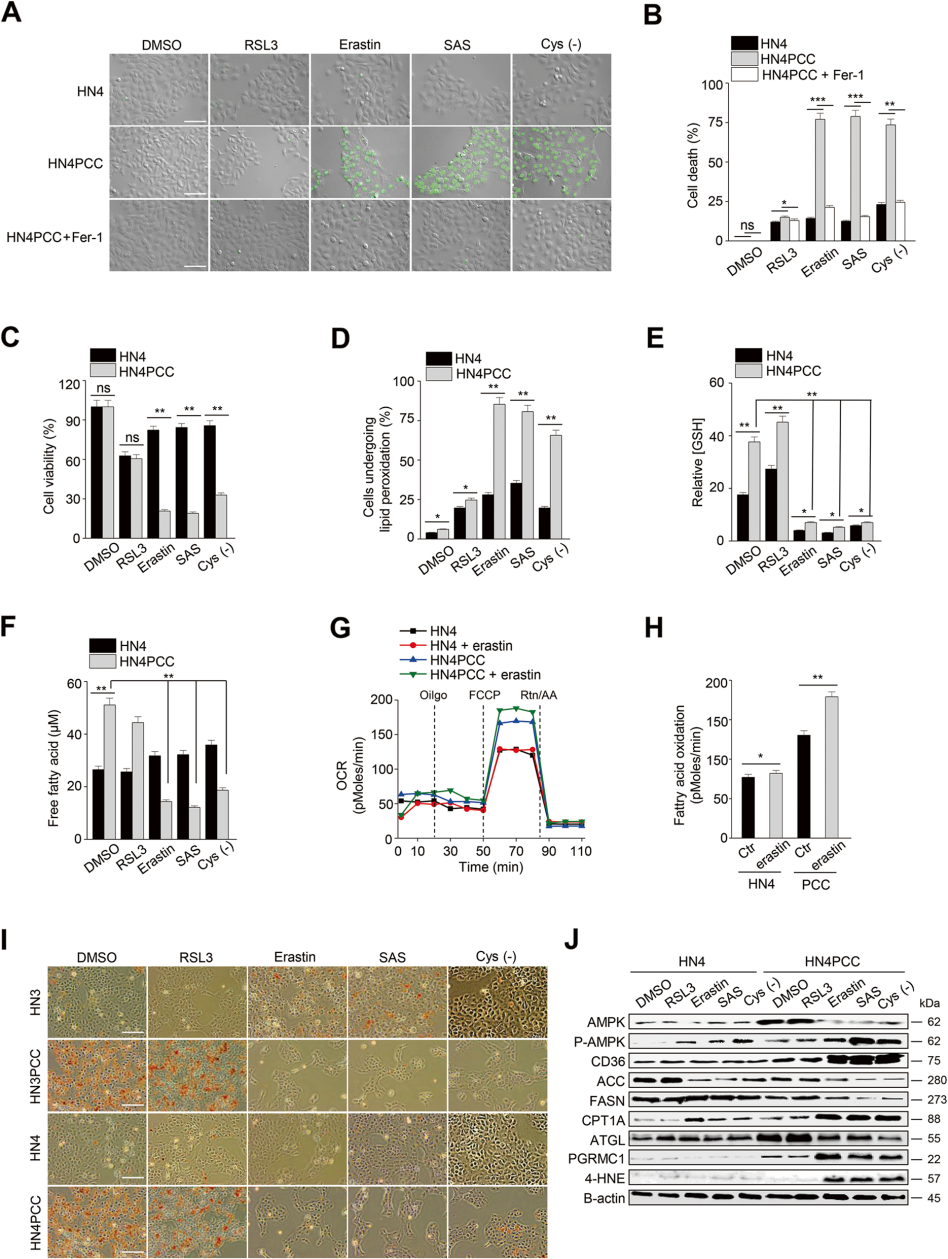
PCC is vulnerable to xCT inhibitors. **A**-**C** Cell death and viability assays in HN4 parental cells and PCC. Cell death was assessed using SYTOX™ Green stain in the cells treated with or without 2 μM ferrostatin-1 plus 1 μM RSL3, 10 μM erastin, 0.5 mM sulfasalazine (SAS), or cyst(e)ine deprivation for 48 h. Dead cells were quantified by counting SYTOX Green positive cells. Cell viability was examined using a CCK-8 assay. Scale bar 100 μm. Data are means and s.d. from three technical replicates. ns, non-significance; *P < 0.05, **P < 0.01, ***P < 0.001 relative to PCC. **D** Lipid peroxidation was examined using BODIPY™ C11 and fluorescence-activated cell sorting (FACS) in parental cells and PCC after exposure to the ferroptosis inducers of 1 μM RSL3, 10 μM erastin, 0.5 mM SAS, and cyst(e)ine deprivation for 8 h. *P < 0.05, **P < 0.01 relative to parental cells. **E** and **F** Relative glutathione (GSH) and free fatty acid contents in parental cells and PCC after treatment with ferroptosis inducers for 24 h; 1 μM RSL3, 10 μM erastin, 0.5 mM SAS, or cyst(e)ine deprivation. *P < 0.05, **P < 0.01 relative to parental cells. **G** and **H** FAO in parental cells and PCC with or without 10 μM erastin was quantified via assessing changes in OCR when exposed to etomoxir or not. *P < 0.05, **P < 0.01 relative to parental cells. **I** and **J** Oil red O staining and immunoblotting in parental cells and PCC after treatment with ferroptosis inducers for 24 h; 1 μM RSL3, 10 μM erastin, 0.5 mM SAS, and cyst(e)ine deprivation. Scale bar 100 μm

Cell death significantly increased in PCC compared to parental cells when exposed to ferroptosis inducers inhibiting xCT: erastin, sulfasalazine, and cyst(e)ine deprivation (*P* < 0.01) (Fig. 2A–B). The cells showed minimal sensitivity to RSL3, an inhibitor of GPX4. Cell death by ferroptosis inducers was recovered by co-treatment with ferrostatin-1, an inhibitor of ferroptosis. Cell viability by xCT inhibitors also significantly decreased in PCC (*P* < 0.01) (Fig. 2C). Lipid peroxidation more increased in PCC than in parental cells when exposed to the xCT inhibitors (*P* < 0.01) (Figs. 2D and S1C). Cellular glutathione levels in PCC were higher than parental cells and were significantly decreased by xCT inhibitors (*P* < 0.01) (Fig. 2E). Free fatty acid and FAO levels increased in PCC were significantly decreased by xCT inhibitors (*P* < 0.01) (Fig. 2F–H). Lipid deposition in PCC was significantly decreased by xCT inhibitors (Fig. 2I). On immunoblotting, pAMPK, CD36, CPT1A, and 4-HNE increased in PCC but not in parental cells when exposed to the xCT inhibitors, whereas ACC, FASN, and ATGL decreased in PCC (Fig. 2J). These were chosen due to their relations to autophagy, lipophagy, FAS, FAO, and lipid peroxidation. Taken together, PCC showed more vulnerability to xCT inhibitors than parental cells.

### Regulation of FAO or FAS in PCC modestly increases ferroptosis

PCC showed significant changes in fatty acid metabolism by shifting to FAO. Therefore, we examined whether modulation of fatty acid metabolism related to FAO or FAS affected ferroptosis sensitivity by the ferroptosis inducers. CPT1A is a rate-limiting enzyme for mitochondrial FAO that can be blocked by shCPT1A transfection into cells or etomoxir, a small-molecule inhibitor of CPT1A [20]. Malonyl-CoA is formed from citrate by acetyl-CoA carboxylases (ACC), and perphenazine activates protein phosphatase 2 (PP2A), converting ACC to an active form [21]. All the malonyl-CoA, citrate, and perphenazine can increase FAS in cells. The inhibition of CPT1A or activation of ACC modestly increased ferroptotic cell death in PCC with the treatment of RSL3 or erastin (Fig. S2A–C). Lipid peroxidation and mitochondrial ROS generation modestly increased in PCC treated with RSL3 or erastin (Fig. S2D–F). CPT1A silencing inhibited FAO, but enhanced FAS by the increased expression of ACC, FASN, and CD36, and malonyl-CoA, citrate, or perphenazine treatment also increased FAS by the increased expression of CD36 or ACC and FASN (Fig. S3A–B). Expression of PGRMC1, ATGL, and LN3B was not significantly changed. Free fatty acid, lipid droplets, and lipid deposition were modestly increased by inhibiting FAO and activating FAS, which were modestly decreased by erastin treatment (Fig. S3D–F). Inhibition of FAO and activation of FAS increased the expression of ACSL4, 4-HNE, pAMPK, LC3B, p16, and α-tubulin to minimal levels (Fig. S4A–B). Lipid droplets were minimally changed by CTP1A inhibition or citrate treatment combined with treatment of erastin (Fig. S4C–D). Taken together, inhibition of FAO and activation of FAS had a modest effect on ferroptosis sensitivity and minimal impact on lipophagy.

### PGRMC1 is a critical regulator of enhanced ferroptosis in PCC

**Fig. 3:**
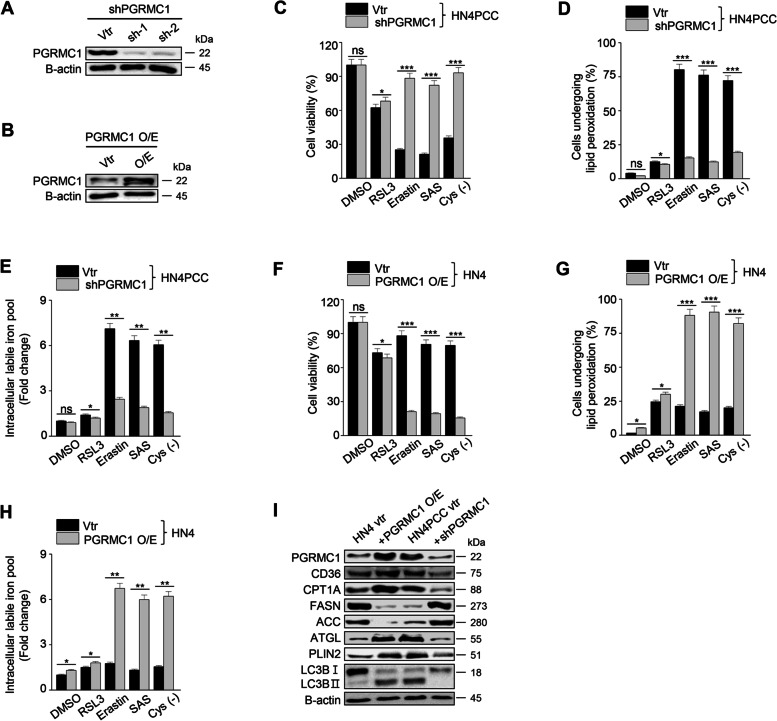
PGRMC1 expression induces ferroptosis. **A** and **B** Immunoblotting in HN4PCC with vector or shPGRMC1 transfection (**A**) and HN4 parental cells with control or PGRMC1 overexpression vector transfection (**B**). **C**-**E** Cell viability, lipid peroxidation, and labile iron pool in the PCC with vector or shPGRMC1. The cells were examined after treatment with ferroptosis inducers: 1 μM RSL3, 10 μM erastin, 0.5 mM SAS, and cyst(e)ine deprivation for 48 h for cell viability and 8 h for lipid peroxidation and labile iron pool. Data are means and s.d. from three technical replicates. *P < 0.05, **P < 0.01, ***P < 0.001 relative to vector control (vtr). **F**-**H** Cell viability, lipid peroxidation, and labile iron pool in HN4 parental cells and PGRMC1 overexpression cells were detected after treatment with ferroptosis inducers: 1 μM RSL3, 10 μM erastin, 0.5 mM SAS. *P < 0.05, **P < 0.01, ***P < 0.001 relative to vector control. **I** Immunoblotting in HN4 parental cells with control or PGRMC1 overexpression vector transfection and HN4PCC with vector or shPGRMC1 transfection

We identified that PGRMC1 expression robustly increased in PCC. PGRMC1 is known to alter lipid metabolism and promote the proliferation and progression of cancer cells [22]. Next, we inhibited PGRMC1 expression in PCC by transfection of shPGRMC1 vector and induced PGRMC1 expression in parental HNC cells by transfection of PGRMC1 overexpression vector (Fig. 3A–B). PGRMC1 silencing in PCC significantly decreased ferroptosis sensitivity by xCT inhibitors in examining cell viability, death, and labile iron pool (Fig. 3C–E). Conversely, PCRMC1 overexpression in parental HNC cells significantly decreased cell viability and increased cell death, lipid peroxidation, and intracellular labile iron pool when the cells underwent xCT inhibitors (Fig. 3F–H). On immunoblotting, CD36, CPT1A, ATGL, PLIN2, LC3B, and PGRMC1 expression increased in parental cells with PGRMC1 overexpression as the same as shown in PCC, which were reversed in PCC with shPGRMC1 transfection (Fig. 3I). Therefore, our data showed that PGRMC1 expression was closely related to ferroptosis sensitivity to xCT inhibitors.

### PGRMC1 promotes ferroptosis via lipophagy and tubulin detyrosination

**Fig. 4:**
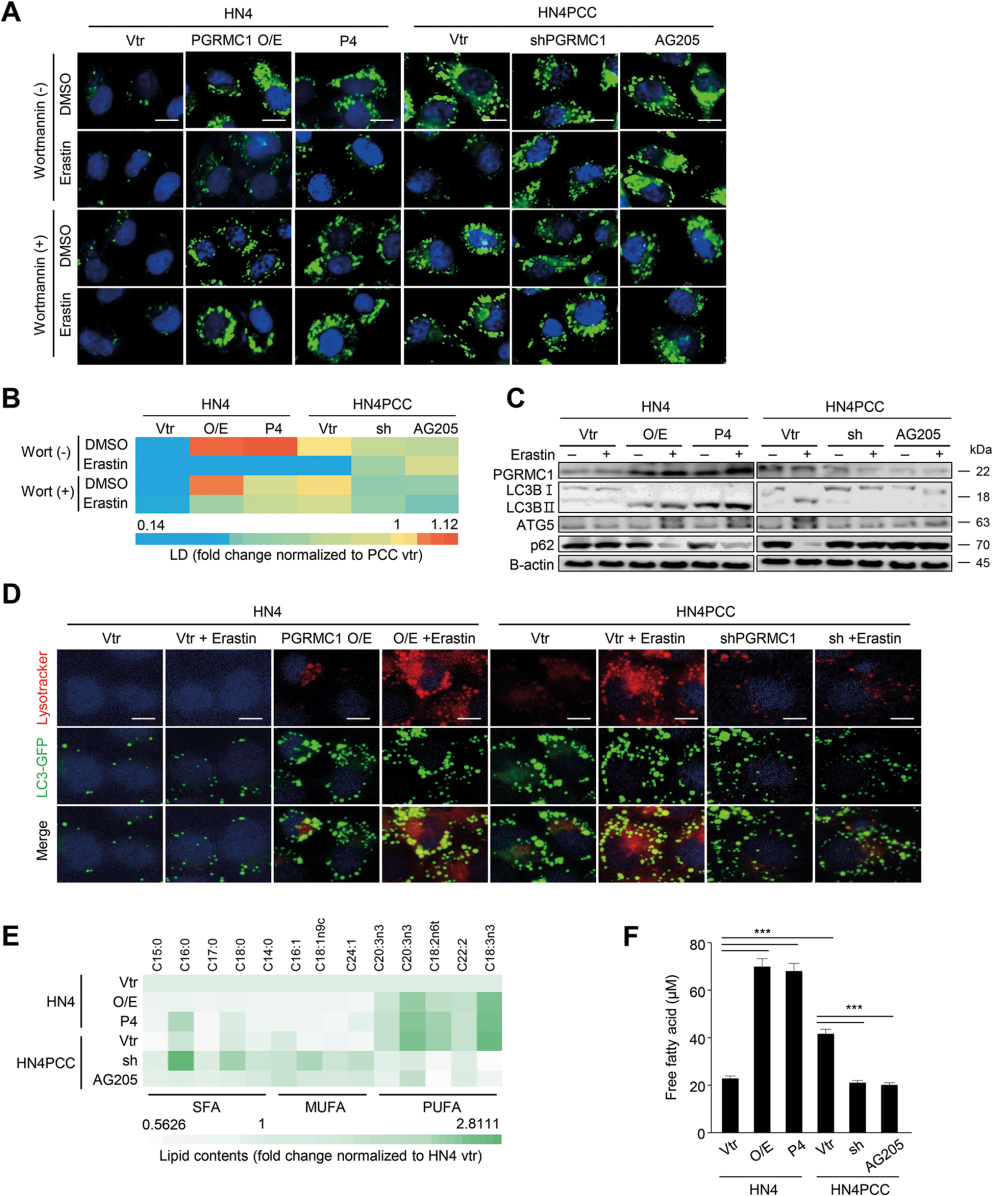
PGRMC1 promotes ferroptosis via lipophagy. **A** and **B** Lipid droplets in HN4 parental cells and PCC with or without erastin treatment. HN4 cells were transfected with vector (vtr) or PGRMC1 overexpression (O/E) vector or treated with 100 nM progesterone (P4). HN4PCC were transfected with vector or shPGRMC1 or treated with 20 μM AG205, a PGRMC1 antagonist. The cells were treated with or without 30 nM Wortmannin plus DMSO or 10 μM erastin for 24 h. Lipid droplets (green) were quantified using ImageJ, displayed as a heatmap relative to PCC vector control. Scale bar 10 μm. **C** Immunoblotting in HN4 parental cells and PCC with or without PGRMC1 overexpression or inhibition and with DMSO or 10 μM erastin treatment for 24 h. **D** Co-staining of Lysotracker™ Deep Red and LC3-GFP (green) in HN4 parental cells and PCC with or without PGRMC1 overexpression or inhibition and with DMSO or 10 μM erastin for 4 h. Nuclei (blue) were stained with DAPI. Scale bar 10 μm. **E** and **F** Quantification of cellular lipid contents by gas chromatography and mass spectrometer (GC-MS) and of free fatty acids in HN4 parental cells and PCC with or without PGRMC1 overexpression or inhibition. The GC-MS data were normalized to HN4 vector control. O/E, PGRMC1 overexpression vector; sh, shPGRMC1; SFA, saturated fatty acids; MUFA, monounsaturated fatty acids; PUFA, polyunsaturated fatty acids. Data are means and s.d. from three technical replicates. ***P < 0.001 relative vector control

PGRMC1 can promote autophagy by elevating cleaved LC3B levels [14]. Therefore, we examined whether PGRMC1 engaged in cytosolic lipophagy formation in cancer cells with PGRMC1 expression when exposed to ferroptosis inducers. Erastin treatment induced lipid droplet degradation by autophagy in PCC or parental cells with transfection of PGRMC1 overexpression vector or progesterone (P4) treatment (Fig. 4A–D). The degradation of lipid droplets by erastin treatment was inhibited by Wortmannin, an autophagy inhibitor. Conversely, erastin treatment minimally affected lipid droplet degradation and autophagosome formation in parental cells with vector transfection or PCC with the inhibition of PGRMC1 expression by transfection of shPGRMC1 vector or AG205 treatment. Cleaved LC3B increased, and p62 decreased along with the autophagy process in the cancer cells with PGRMC1 expression (Fig. 4C). Pharmacological regulation of PGRMC1 expression by P4 or AG205 in parental cells and PCC significantly affected ferroptosis sensitivity by xCT inhibitors in terms of cell viability, lipid peroxidation, and intracellular labile iron pool (*P* < 0.01) (Fig. S5A–F). PGRMC1 expression was also associated with increased autophagosome formation (Fig. 4D). Free fatty acid contents significantly increased in parental cells with PGRMC1 overexpression or P4 treatment in addition to PCC, whereas those significantly decreased in PCC with PGRMC1 inhibition (*P* < 0.001) (Fig. 4F). In particular, PUFA contents increased in parental cells with PGRMC1 overexpression or PCC, reversed by PGRMC1 inhibition (Fig. 4E). Conversely, monounsaturated fatty acid (MUFA) contents decreased in cancer cells with PGRMC1 expression or P4 treatment but increased in PCC with shPGRMC1 vector transfection or AG205 treatment.

**Fig. 5:**
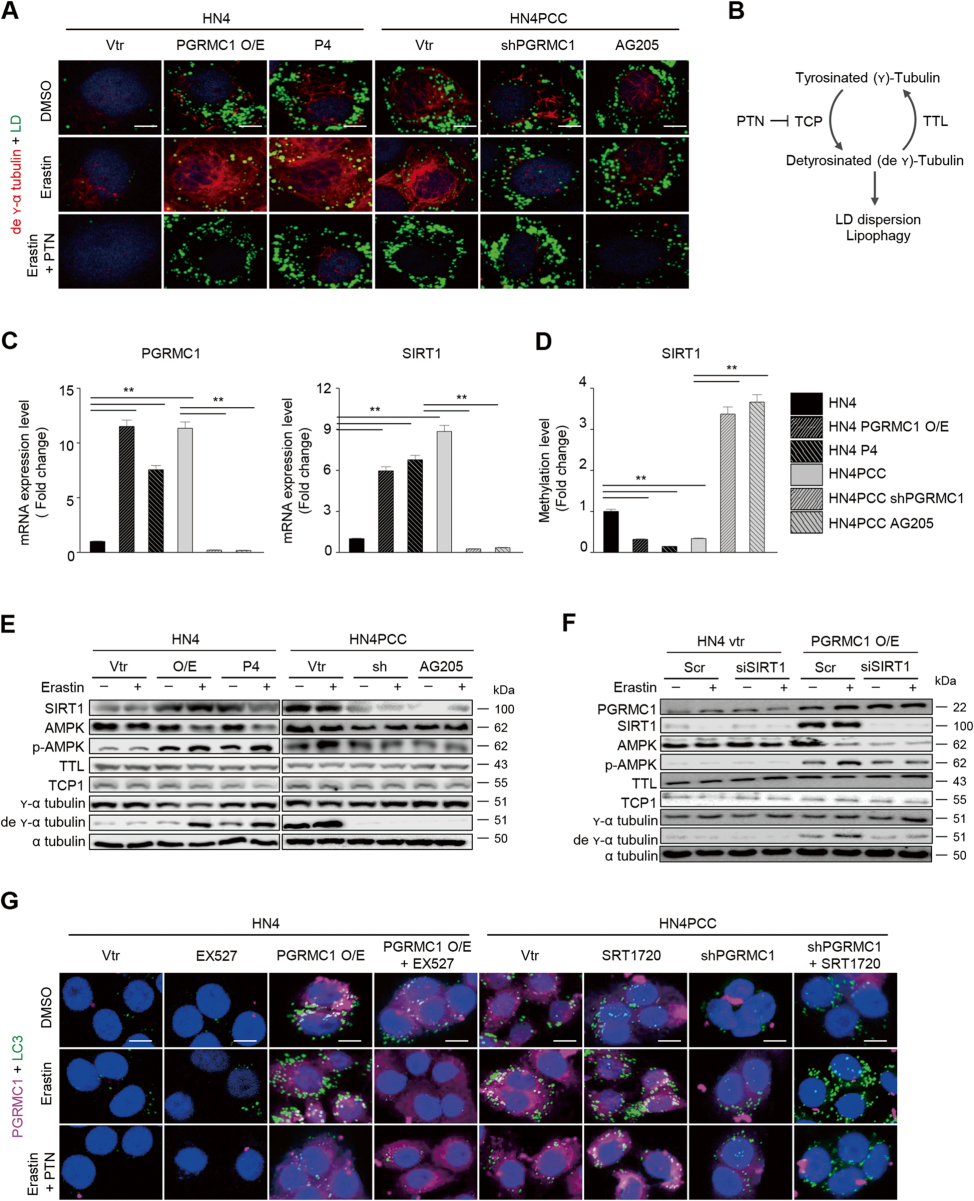
PGRMC1 promotes lipophagy via increased tubulin detyrosination. **A** Co-staining of lipid droplet (LD, green) and detyrosinated α-tubulin (red) in HN4 parental cells and PCC with or without PGRMC1 overexpression or inhibition. HN4 cells were transfected with control or PGRMC1 overexpression vector (vtr) or treated with 100 nM P4. HN4PCC were transfected with vector or shPGRMC1, or 20 μM AG205. The cells were also treated with DMSO, 10 μM erastin, or 10 μM erastin plus 20 μM parthenolide (PTN), a sesquiterpene lactone inhibiting the activity of tubulin carboxypeptidase (TCP) [24], for 24 h. Nuclei (blue) were stained with DAPI. Scale bar 5 μm. **B** An illustration showing the relation between tubulin detyrosination and lipophagy. **C** and **D** mRNA expression (**C**) and genomic DNA methylation levels (**D**) in HN4 parental cells and PCC with or without PGRMC1 overexpression or inhibition. Data are means and s.d. from three technical replicates. **P < 0.01 relative to vector control. **E** and **F** Immunoblotting in HN4 parental cells and PCC with or without PGRMC1 overexpression or inhibition and with DMSO or 10 μM erastin for 24 h. HN4 cells with control or PGRMC1 overexpression vector transfection were also transfected with scrambled or SIRT1 siRNA and treated with DMSO or 10 μM erastin for 24 h (**F**). **G** Co-staining for PGRMC1 (magenta), LC3-GFP (green), and nuclei (blue) in the cells treated with 10 μM EX527 (a selective SIRT1 inhibitor), 5 μM SRT1720 (a selective SIRT1 activator), 10 μM erastin, 20 μM PTN, or their combinations for 24 h. Scale bar 5 μm

Next, we examined whether the detyrosination of α-tubulin by PGRMC1 contributed to lipophagy and ferroptosis sensitivity by xCT inhibitors because PGRMC1 is known to induce microtubule stability [23]. Detyrosinated α-tubulin levels increased with lipid droplets in PCC or parental cells with PGRMC1 overexpression or P4 treatment and decreased in parental cells with vector transfection or PCC with PGRMC1 inhibition (Fig. 5A–B). Erastin treatment significantly increased the levels of detyrosinated α-tubulin and degraded lipid droplets in PGRMC1 expressing or activating cells, which were inhibited by parthenolide (PTN), a sesquiterpene lactone inhibiting the activity of tubulin carboxypeptidases (TCP) [24] (Fig. 5A–B, and E). PGRMC1 affects DNA methylation, including SIRT1 that can mediate ATGL-driven lipophagy/autophagy [25, 26]. SIRT1 is necessary for AMPK activation that promotes lipid droplet dispersion on detyrosinated microtubules [27]. Therefore, these molecular expressions and links were also examined along with PGRMC1 or SIRT1 expression changes. The SIRT1 mRNA and protein levels and pAMPK were elevated along with PGRMC1 presentation (Fig. 5C and E). SIRT1 expression was increased by PGRMC1 overexpression in parental cells, which were silenced by siSIRT1 transfection (Fig. 5F). The methylation levels of SIRT1 were inversely correlated with PGRMC1 or SIRT1 mRNA levels (Fig. 5D). The levels of pAMPK and detyrosinated α-tubulin were significantly increased by erastin treatment, which was reversed by siSIRT1 transfection. However, silencing of SIRT1 did not affect the expression of PGRMC1 (Fig. S6). SIRT1 inhibition in parental cancer cells with PGRMC1 overexpression blocked autophagy, but SIRT1 activation in PCC with PGRMC1 inhibition induced autophagy, which was not affected by parthenolide treatment (Fig. 5G). Therefore, our data showed that PGRMC1 promotes lipophagy and autophagy by increased tubulin detyrosination, which contributed to increased ferroptosis sensitivity in PCC.

### PGRMC1 expression increases FAO and ferroptosis sensitivity in vivo

**Fig. 6:**
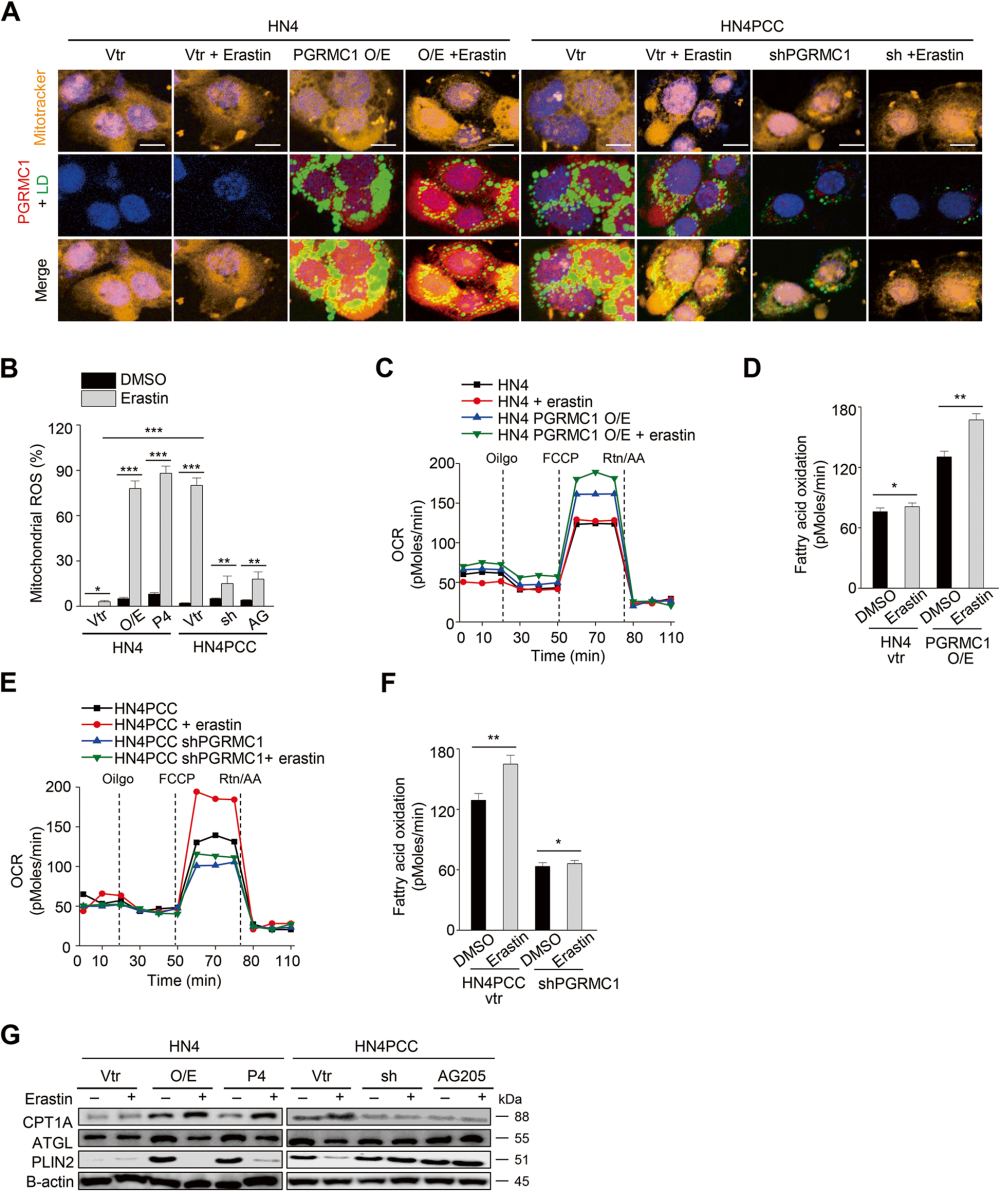
PGRMC1 increases FAO by anchoring lipid droplets to mitochondria. **A** Co-staining of mitotracker (orange), lipid droplets (LD, green), and PGRMC1 (red) in HN4 parental cells and PCC with or without 10 μM erastin treatment for 24 h. HN4 cells were transfected with vector (vtr) or PGRMC1 overexpression vector or treated with 100 nM P4. HN4PCC were transfected with vector or shPGRMC1 or treated with 20 μM AG205. Scale bar 5 μm. **B** Mitochondrial ROS was examined using incubation with 5 μM mitoSOX™ Red and FACS in HN4 parental cells and PCC with or without PGRMC1 overexpression or inhibition and with DMSO or 10 μM erastin for 8 h. Data are means and s.d. from three technical replicates. *P < 0.05, **P < 0.01, ***P < 0.001 relative to DMSO control or between different groups. **C**-**F** Quantification of FAO in HN4 parental cells and PCC with or without PGRMC1 overexpression or inhibition and with DMSO or 10 μM erastin. *P < 0.05, **P < 0.01 relative to DMSO control. **G** Immunoblotting in HN4 parental cells and PCC with or without PGRMC1 overexpression or inhibition and with DMSO or 10 μM erastin for 24 h. O/E, PGRMC1 overexpression vector; sh, shPGRMC1; P4, progesterone; AG, AG205

FAO can be promoted by contact between lipid droplets and mitochondria [28]. Therefore, we examined whether PGRMC1 involved the connection between lipid droplets and mitochondria for increasing FAO in cancer cells. Immunofluorescent staining showed that lipid droplets and mitochondria were co-staining in parental cells with PGRMC1 overexpression or PCC, and lipid droplets were degraded by erastin treatment (Fig. 6A). Mitochondrial ROS and FAO significantly increased in these cells with PGRMC1 expression and were further boosted by erastin treatment, inhibited by PGRMC1 inhibition (Fig. 6B–F). This was accompanied by the changes of CPT1A, ATGL, and PLIN2 expression with or without PGRMC1 expression and erastin treatment (Fig. 6G).

**Fig. 7:**
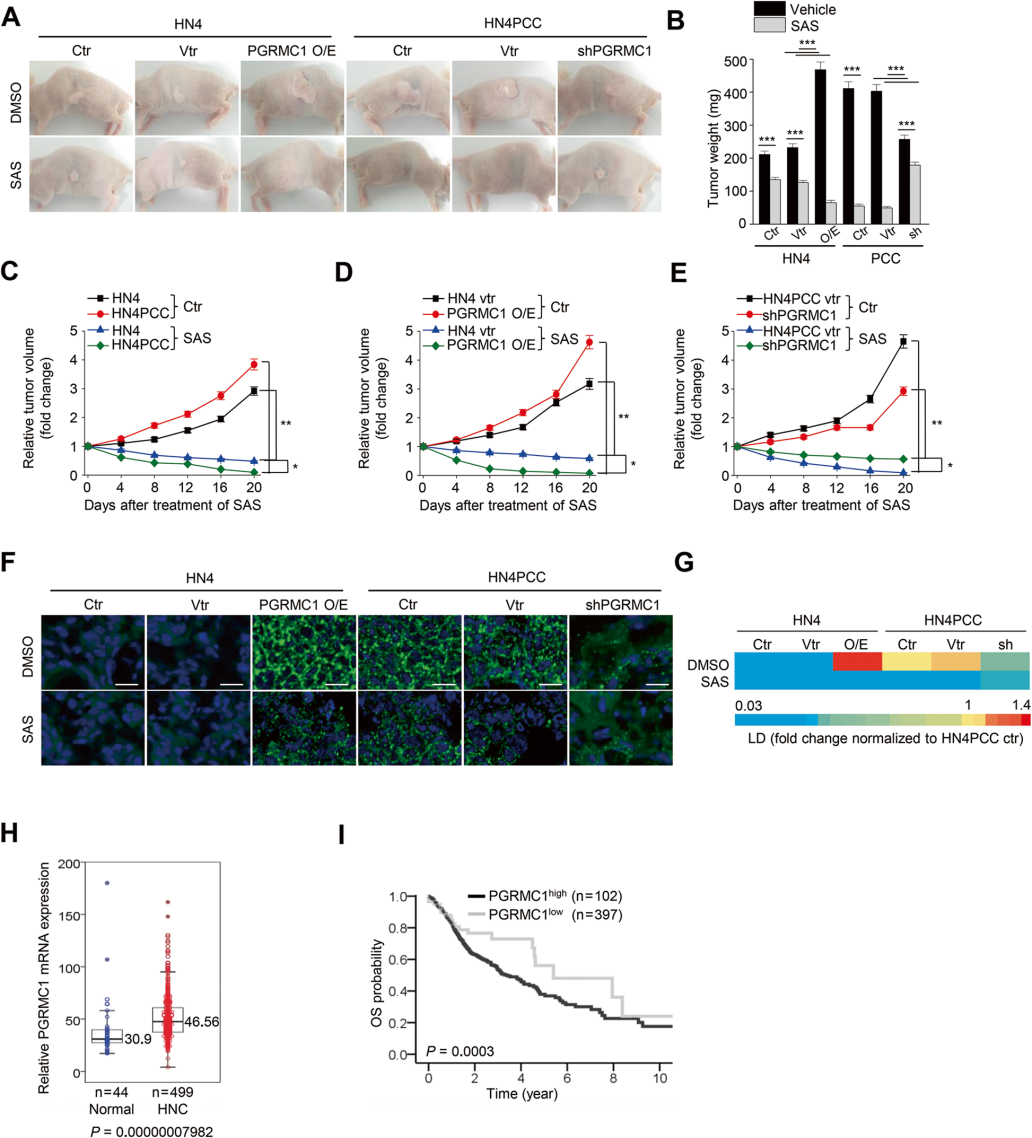
PGRMC1 expression increases ferroptosis sensitivity in vivo. **A**-**E** Representative images, tumor weights, and tumor volumes in HN4 parental cells and PCC with or without vector (vtr), PGRMC1 overexpression, or shPGRMC1 transfection, and vehicle (ctr) or SAS treatment. *P < 0.05, **P < 0.01 relative to vehicle control or between different groups. **F** and **G** Lipid droplet staining of tumors. Lipid droplets (green) were quantified using ImageJ and were normalized to HN4PCC vector control. Scale bar 100 μm. **H** Comparison of PGRMC1 mRNA expression between normal mucosa and tumor samples in the HNC patient cohort from TCGA datasets. *t*-test, *P* < 0.001. **I** Kaplan-Meier curves estimating overall survival (OS) according to patients with low and high expression levels of tumor PGRMC1 mRNA (cutoff = 34.57) from the TCGA datasets. Log-rank test, *P* < 0.001

Animal experiments showed that the in vivo tumor growth was promoted by PGRMC1 expression and was more significantly inhibited in the PGRMC1-expressing tumors by the treatment of sulfasalazine, an xCT inhibitor (Fig. 7A–E). Lipid droplets were more abundant in PGRMC1 overexpression or PCC tumors than low PGRMC1 expression tumors (Fig. 7F–G). Lipid droplets in tumors were reduced by sulfasalazine treatment. From the HNC patient cohort of TCGA datasets, PGRMC1 mRNA expression was significantly higher in HNC samples than normal mucosa (*P* < 0.001) (Fig. 7H). Overall survival was significantly lower in patients with high PGRMC1 expression than those with low PGRMC1 expression (*P* < 0.001) (Fig. 7I). From the TCGA datasets, mRNA expression of PGRMC1 was significantly correlated with those of autophagy-related genes, such as SQSTM1, ATG5, MAP1LC3B, and SIRT1 (all *P* < 0.05) (Table S1).

## Discussion

**Fig. 8:**
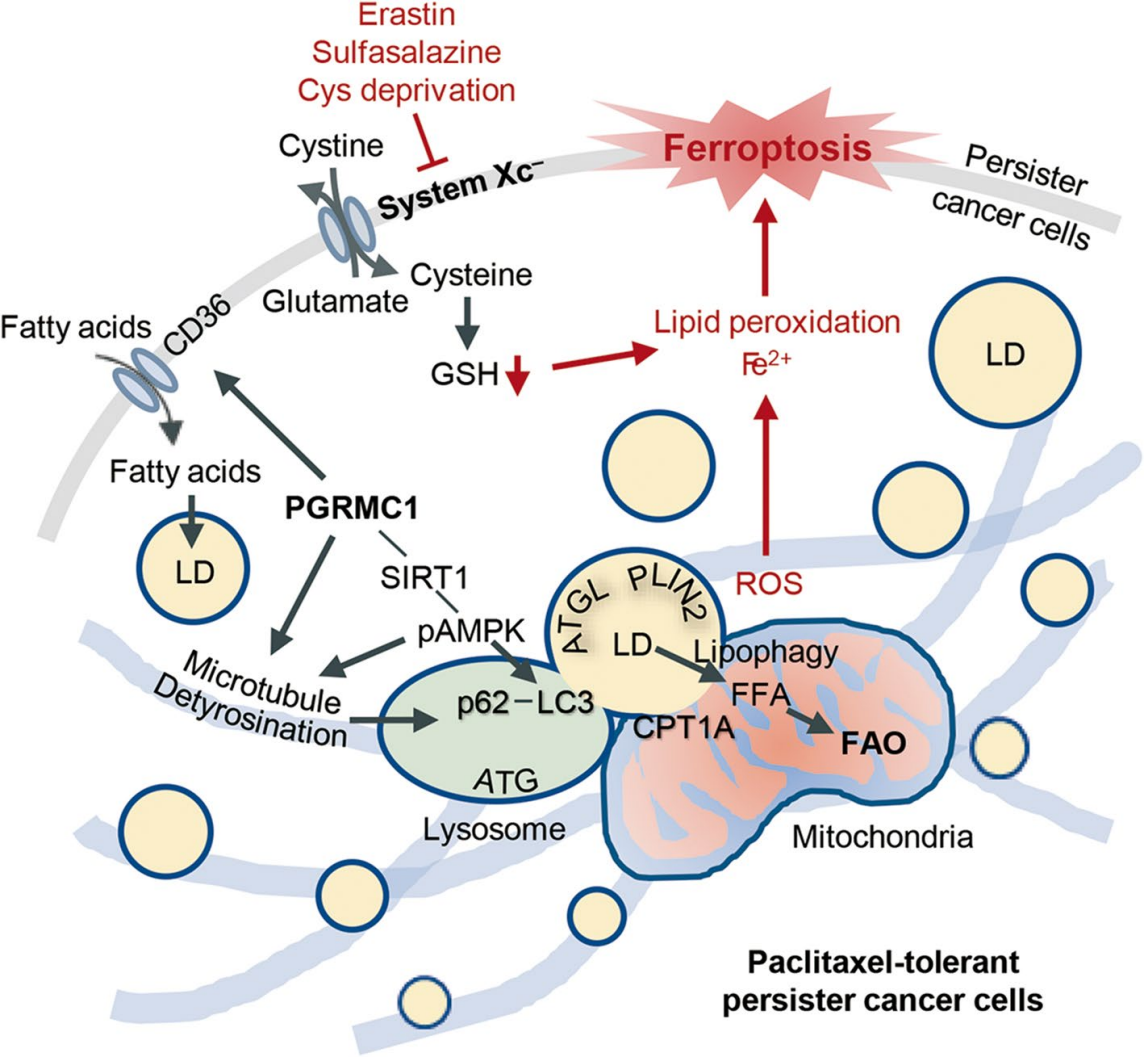
An illustration shows that PGRMC1 expression promotes ferroptosis in PCC. PCC has increased PGRMC1 expression related to increased free fatty acid acids (FFA), lipid droplets (LD), and fatty acid oxidation (FAO). PGRMC1 expression increased FAO and ferroptosis sensitivity by xCT inhibitors via lipophagy and tubulin detyrosination

The present study showed that PCC had PGRMC1 upregulation related to increased free fatty acids, lipid droplets, and FAO. PCC was highly sensitive to ferroptosis inducers inhibiting the xCT, such as erastin, sulfasalazine, and cyst(e)ine deprivation, but less sensitive to GPX4 inhibition by RSL3. Regulation of FAO or FAS in PCC modestly increases ferroptosis. Ferroptosis sensitivity to xCT inhibitors was reduced by PGRMC1 silencing in PCC and increased by PGRMC1 overexpression in parental cells. Lipid droplets from xCT inhibitors in PCC were substantially degraded by autophagic processes characterized as lipophagy and interaction with the mitochondria. This was facilitated by the increased tubulin detyrosination that was raised by SIRT1 activation but decreased by SIRT1 inhibition (Fig. 8). PGRMC1 expression elevated FAO and ferroptosis sensitivity from in vivo mice experiments with tumor transplantation. Therefore, our data suggest that PGRMC1 expression was characterized as the chemo-resistant property of PCC that was vulnerable to ferroptosis by xCT inhibitors.

PCC showed a metabolic shift to FAO along with PGRMC1 upregulation. Alteration of cellular metabolism and lipid homeostasis mediates the chemoresistance of tumor cells to anti-cancer drugs [22]. Enhanced glutaminolysis increases the cellular levels of glutamine, glutamate, and GSH in breast cancer cells treated with paclitaxel [29]. Adipocyte and lipid metabolic reprogramming is a hallmark of resistant cancer cells: it stores excessive energy as lipid droplets that can be broken down free fatty acids and transported into the mitochondria through accelerated FAO [30]. Paclitaxel induces cytotoxicity in tumor cells by binding to tubulin, stabilizing the microtubule, preventing its disassembly, and selectively arresting the cell cycle in the G2/M phase [31]. Predictably, the disruption of microtubule dynamics alters the binding of paclitaxel to the microtubule, resulting in paclitaxel insensitivity in cancer cells [32]. PGRMC1 also directly interacts with tubulin, stabilizes the spindle microtubule, and affects mitosis through a microtubule-dependent process [23]. PGRMC1 expression promoted tumor growth and decreased the chemosensitivity of human xenograft tumors to paclitaxel [33]. The findings may hint at the potential link between paclitaxel insensitivity and PGRMC1. Our data also supported that paclitaxel insensitivity in PCC was associated with the altered lipid metabolism and disoriented microtubule by its decreased stabilization, accompanied by the increased expression of PGRMC1.

PCC was quite sensitive to ferroptosis inducers. Ferroptosis is a new form of necrosis based on the iron-dependent accumulation of excessive lipid peroxidation [10]. Accumulation of free fatty acids, PUFAs, is an essential prerequisite to producing lipid peroxidation by the phospholipid-binding of ROS from iron-mediated reactions of peroxides [8]. Altered lipid reprogramming characterized by increased free fatty acids, lipid droplets, and FAO in PCC may contribute more vulnerability to ferroptosis than parental cells. Therapy-resistant persister cancer cells show a high-mesenchymal cell state and dependency on a lipid peroxidase pathway that causes the vulnerability to ferroptotic cell death [5]. This cell state involves the alteration of lipid metabolism characterized by the increased activity of enzymes that promote PUFA synthesis and the high expression of ZEB1 that induces EMT in epithelial cancers [4]. Inhibition of a lipid peroxidase pathway GPX4 induces ferroptosis in therapy-resistant cancer cells and prevents tumor relapse in vivo [5]. PCC was also vulnerable to ferroptotic cell death in the present study, mainly induced by the xCT inhibitors. The enhanced ferroptosis sensitivity might be in part explained by the increased free fatty acids and PUFA contents, lipid droplets, and FAO in PCC. However, ferroptosis was only modestly promoted by the regulation of FAO or FAS in PCC. Besides, there were some differences in ferroptosis sensitivity between xCT and GPX4 inhibitors. This needed to elucidate further mechanisms that explained the increased ferroptosis sensitivity in PCC.

The present study showed that PGRMC1 involved lipophagy by increased tubulin detyrosination and interaction with the mitochondria. PGRMC1 involved the substantial degradation of lipid droplets along with the autophagic process of autophagosome formation and lipophagy. PGRMC1 promotes autophagy via direct binding to a critical component of the autophagy machinery LC3, causing increased cleaved LC3 levels and the degradative activity of autophagy [14]. Lipophagy is an autophagic process targeting lipid droplets to break down in lysosomes that are degraded by promoting interaction between autophagosomal lipid droplets and lysosomes [34]. Lipid droplet dispersion can be involved in the lipophagic process on the detyrosinated microtubule to increase mitochondrial FAO via AMPK activation [27]. Lipid droplet lipolysis and autophagy move cytosolic fatty acids from lipid droplets to mitochondria via the fusion of lipid droplets and mitochondria [35]. Mitochondria fusion with lipid droplets induces lipid storage and utilization, resulting in simultaneous involvement in FAO and lipid droplets formation in cells [36]. Detyrosinated microtubules spatially interact with lysosomes, which increases autolysosomes, a fusion intermediate of autophagosomes and lysosomes. These play a role in carrying lipid droplets to break down in lysosomes and their utilization for mitochondrial FAO [37]. Besides, PGRMC1 expression alters genomic CpG methylation, such as NAD-dependent deacetylases, e.g., sirtuins [25]. SIRT1 activation might promote autophagy/lipophagy of lipid droplets via AMPK activation via cytosolic lipases such as ATGL [26]. Therefore, our data may support that PGRMC1-dependent lipophagy can be mediated by a SIRT1-AMPK axis.

The present study has shown that PGRMC1 expression was a critical regulator of promoting ferroptosis in PCC. However, this study has several limitations. First, PGRMC1 plays diverse roles in regulating cytochrome P450, steroidogenesis, progesterone signaling, membrane trafficking, mitotic spindle and cell cycle regulation, and autophagy [12]. PGRMC1 may affect the functions of both normal and cancer cells, which might induce ferroptotic cell death in both. Second, PCC showed high sensitivity to xCT inhibitors but relatively more minor sensitivity to GPX4 inhibitors. The current study has not fully elucidated key molecules or pathways that explain different sensitivities between xCT and GPX4 inhibitors. This might be a phenomenon localized in PCC and is dependent on GPX4 expression inhibited by cyst(e)ine metabolism [38]. This needs to be elucidated by further studies. Nonetheless, PGRMC1 expression in PCC promoted the sensitivity to ferroptosis inducers, which might be a promising strategy to eradicate persister cells surviving after conventional or targeted chemotherapy.

## Conclusion

The present study suggests that PGRMC1 is required for xCT inhibitors to induce ferroptosis in PCC. PGRMC1 is a crucial regulator of being more vulnerable to ferroptosis in PCC than parental cells via lipophagy-dependent degradation of lipid droplets and mitochondrial FAO. Activation of PGRMC1 might be a potential strategy to kill recalcitrant cancer cells via promoting their ferroptosis susceptibility.

## Supplementary Information

**Additional file 1: Figure S1.** Paclitaxel-tolerant persister cancer cells (PCC) are vulnerable to xCT inhibitors. **Figure S2.** Regulation of fatty acid metabolism in PCC modestly increases ferroptosis. **Figure S3.** FAO or FAS regulation in PCC modestly increases cellular fatty acids. **Figure S4.** FAO or FAS regulation has minimal effect on lipophagy in PCC. **Figure S5.** PGRMC1 expression is related to ferroptosis sensitivity. **Figure S6.** Immunoblotting in HN4 PCC with scrambled or SIRT1 siRNA transfection and then treated with DMSO or 10 μM erastin for 24 h. **Table S1.** Correlation of mRNA expression levels between PGRMC1 and other genes from the HNC datasets of TCGA.

## Data Availability

All data generated or analyzed during this study are included in the manuscript and its supplementary information files.

## Abbreviations

CCK-8: Cell counting kit-8
DMSO: Dimethyl sulfoxide
EMT: Epithelial-mesenchymal transition
FFA: Free fatty acid
FAO: Fatty acid oxidation
FAS: Fatty acid synthesis
GSH: Glutathione
GPX4: Glutathione peroxidase 4
HNC: Head and neck cancer
4-HNE: 4-hydroxynonenal
LC3: Microtubule-associated proteins 1 light chain 3
LIP: Labile iron pool
15-LOX, ALOX15: 15-lipoxygenase
MUFA: Monounsaturated fatty acid
pAMPK: Phospho-5′ adenosine monophosphate-activated protein kinase
P4: Progesterone
PCC: Paclitaxel-tolerant persister cancer cells
PGRMC1: Progesterone receptor membrane component 1
PUFA: Polyunsaturated fatty acid
ROS: Reactive oxygen species
sirtuin 1: SIRT1
xCT: System xc^−^ cystine/glutamate antiporter
