# Supplementary material for: PGRMC1-dependent lipophagy promotes ferroptosis in paclitaxel-tolerant persister cancer cells
Source: J Exp Clin Cancer Res. 2021 Nov 8;40:350. doi: 10.1186/s13046-021-02168-2 (PMC8573965; doi:10.1186/s13046-021-02168-2)
Supplement: Supplementary file 1 — Additional file 1: Figure S1. Paclitaxel-tolerant persister cancer cells (PCC) are vulnerable to xCT inhibitors. Figure S2. Regulation of fatty acid metabolism in PCC modestly increases ferroptosis. Figure S3. FAO or FAS regulation in PCC modestly increases cellular fatty acids. Figure S4. FAO or FAS regulation has minimal effect on lipophagy in PCC. Figure S5. PGRMC1 expression is related to ferroptosis sensitivity. Figure S6. Immunoblotting in HN4 PCC with scrambled or SIRT1 siRNA transfection and then treated with DMSO or 10 μM erastin for 24 h. Table S1. Correlation of mRNA expression levels between PGRMC1 and other genes from the HNC datasets of TCGA. [file 13046_2021_2168_MOESM1_ESM.docx]

**Supplementary Data**


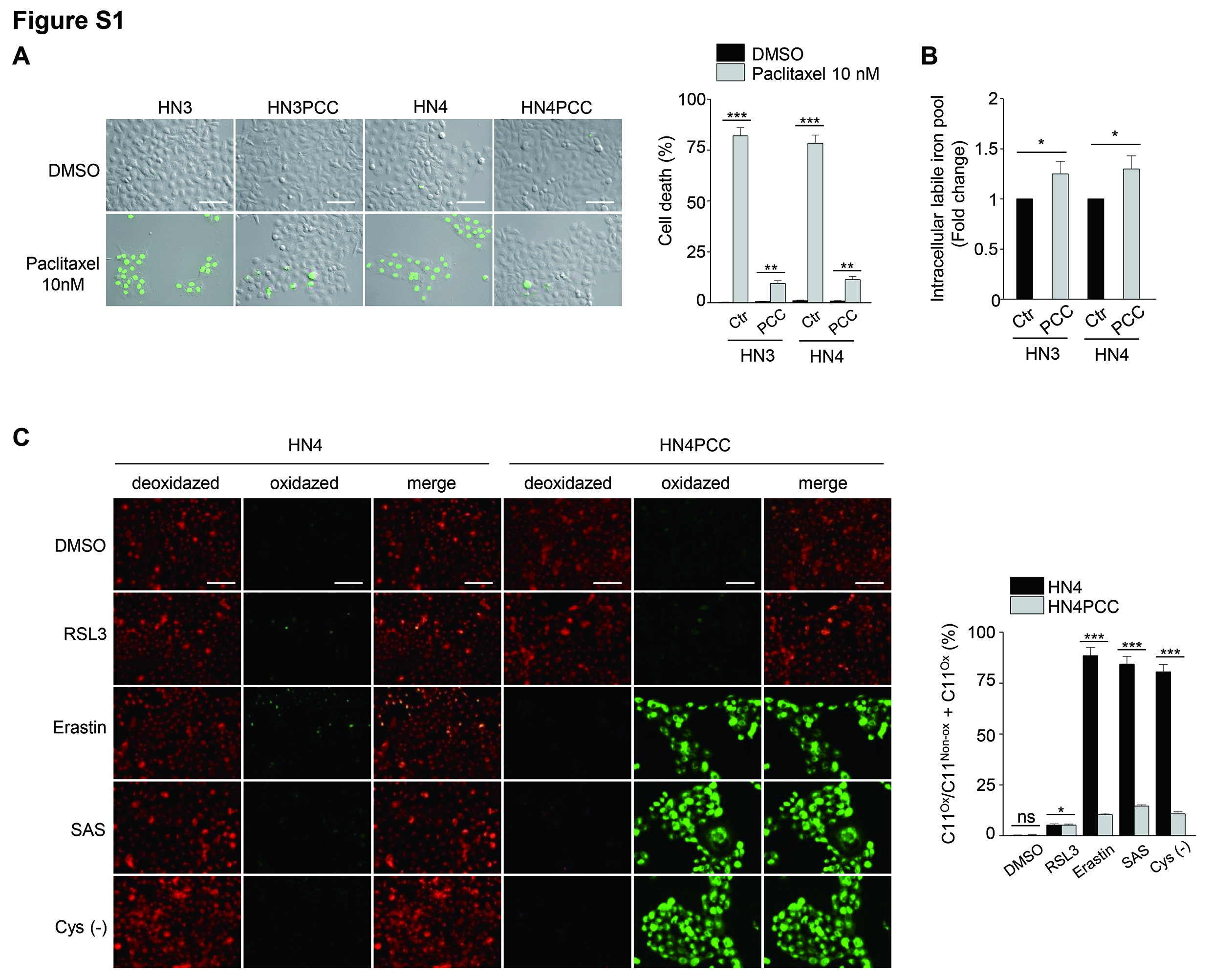


**Figure S1.** Paclitaxel-tolerant persister cancer cells (PCC) are vulnerable to xCT inhibitors. (***A***) Cell death assay in HN3 and HN4 parental cells and PCC with or without 10 nM paclitaxel treatment for 48 h. Cell death was assessed using SYTOX^TM^ Green stain and quantified by counting SYTOX Green positive cells. Scale bar 100 μm. Data are means and s.d. from three technical replicates. ns, non-significance; ***P* < 0.01, ****P* < 0.001 relative to parental cells. (***B***) Labile iron pool in HN3 and HN4 parental cells and PCC. **P* < 0.05. (***C***) The fluorescence images by BOPDIPY^TM^ 581/591 C11 staining. Non-oxidized (red) and oxidized (green) were measured in HN4 parent cells and PCC with or without exposure to the ferroptosis inducers of 1 µM RSL3, 10 µM erastin, 0.5 mM sulfasalazine (SAS), or cyst(e)ine deprivation for 8 h, and then add 5 μM BODIPY C11. Scale bar 100 μm. ns, non-significance; **P* < 0.05 , ****P* < 0.001 relative to parental cells.


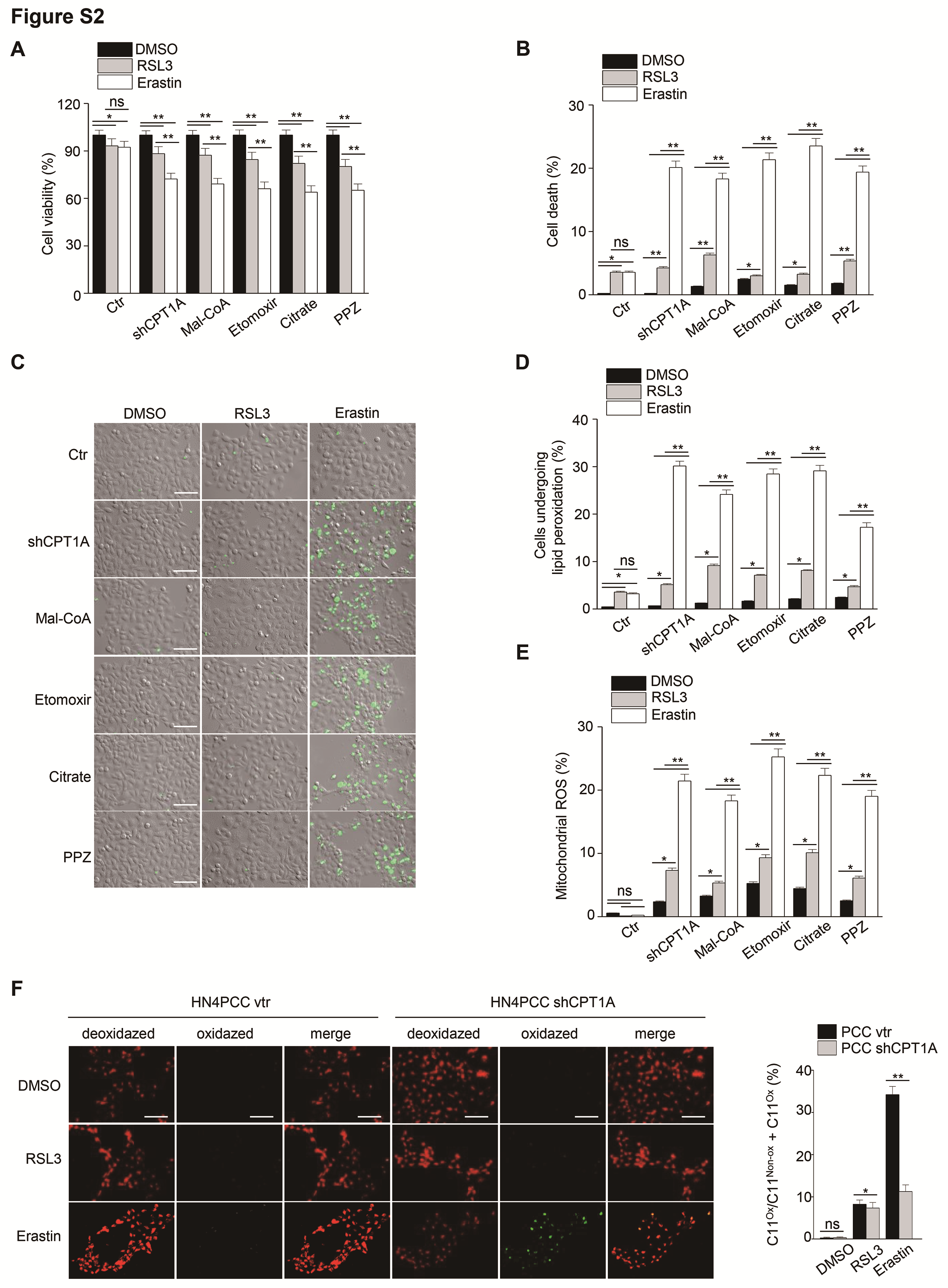


**Figure S2.** Regulation of fatty acid metabolism in PCC modestly increases ferroptosis. (***A***–***C***) Cell viability and death assays in HN4PCC with shCPT1A transfection, or treatment with fatty acid oxidation (FAO) and fatty acid synthesis (FAS) regulating agents. The cells were treated with 50 µM malonyl-CoA, 50 µM etomoxir, 2 mM citrate, or 20 µM perphenazine (PPZ) and co-treated with DMSO or 1 µM RSL3 or 5 µM erastin for 48 h. Cell death was assessed using SYTOX^TM^ Green stain and quantified by counting SYTOX Green positive cells. Cell viability was examined using a CCK-8 assay. Scale bar 100 μm. Data are means and s.d. from three technical replicates. ns, non-significance; **P* < 0.05 , ***P* < 0.01 relative to DMSO or RSL3 treatment. (***D***–***E***) Lipid peroxidation and mitochondrial ROS in HN4PCC with FAO or FAS regulation combined with ferroptosis inducers for 8 h; 1 µM RSL3 and 5 µM erastin. Lipid peroxidation was examined using BODIPY^TM^ C11 and fluorescence-activated cell sorting (FACS). Mitochondrial ROS were examined using incubation with 5 μM MitoSOX^TM^ Red and FACS. **P* < 0.05, ***P* < 0.01 relative to DMSO or RSL3 treatment. (***F***) The fluorescence images by BOPDIPY^TM^ C11 staining in HN4PCC with vector (vtr) or shCPT1A transfection and treatment with DMSO, 1 µM RSL3, or 5 µM erastin for 8 h. Non-oxidized and oxidized forms were stained as red and green colors, respectively. Scale bar 100 μm. **P* < 0.05, ***P* < 0.01 relative to vector control.


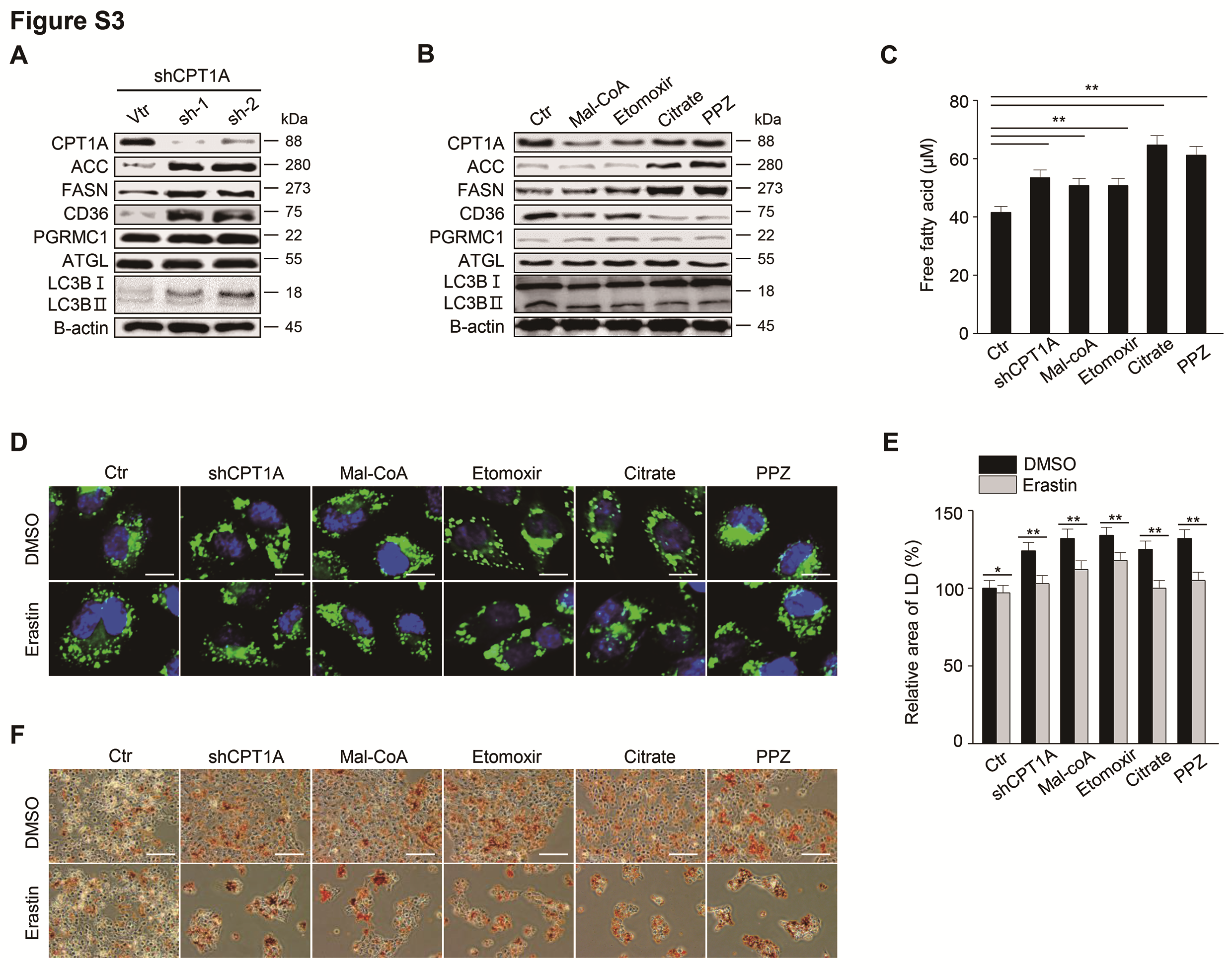


**Figure S3.** FAO or FAS regulation in PCC modestly increases cellular fatty acids. (***A***–***B***) Immunoblotting was examined in HN4PCC with shCPT1A transfection or treatment of FAO or FAS regulating agents. The cells were harvested after 50 µM malonyl-CoA, 50 µM etomoxir, 2 mM citrate, 20 µM perphenazine (PPZ), or DMSO control (ctr) for 24 h. (***C***) Free fatty acids were measured in the cells treated with the indicated agents for 24 h. Data are means and s.d. and from three technical replicates. ***P* < 0.01 relative to PCC. (***D***–***E***) Lipid droplets were stained in the cells with or without FAO and FAS regulating agents after treatment with 5 µM erastin for 24 h. Nuclei (blue) were stained with 4',6-diamidino-2-phenylindole (DAPI). Lipid droplets were quantified using ImageJ. Scale bar 10 μm. **P* < 0.05, ***P* < 0.01 between DMSO and erastin treated groups. (***F***) Oil red O staining in the cells with or without FAO or FAS regulation after treatment with or without 5 µM erastin for 24 h. Scale bar 100 μm.


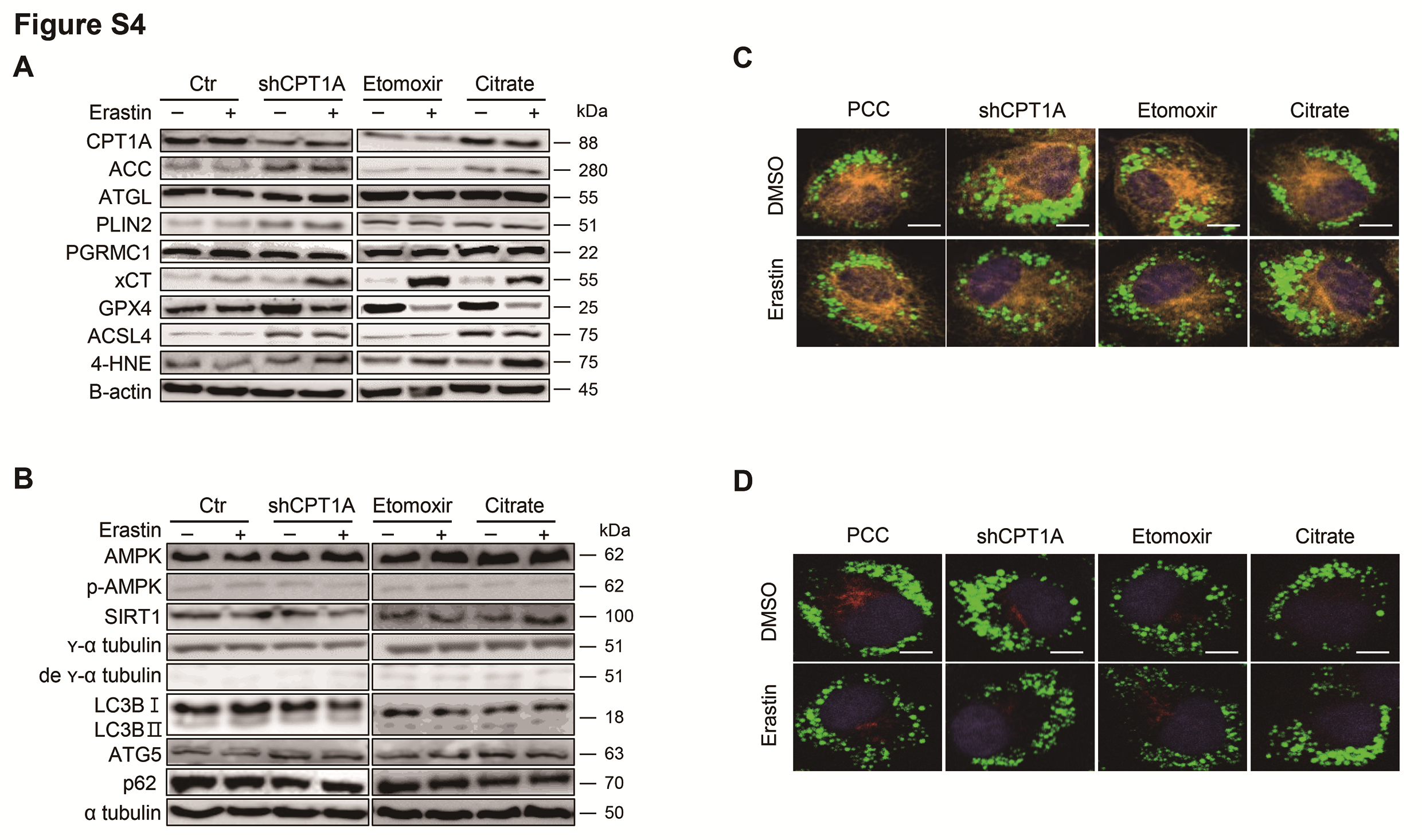


**Figure S4.** FAO or FAS regulation has minimal effect on lipophagy in PCC. (***A***–***B***) Immunoblotting in HN4PCC with shCPT1A transfection or treatment of FAO or FAS regulating agents. The cells were harvested after 50 µM etomoxir, 2 mM citrate, or non-treatment control (ctr) and co-treatment with DMSO or 5 µM erastin for 24 h. (***C***–***D***) Co-immunostaining for α-tubulin (orange, C) or detyrosinated α -tubulin (red, D), and lipid droplets (green) in HN4PCC with shCPT1A transfection or treatment of FAO or FAS regulating agents and co-treatment of DMSO or 5 µM erastin for 24 h. Nuclei (blue) were stained with DAPI. Scale bar 5 μm.


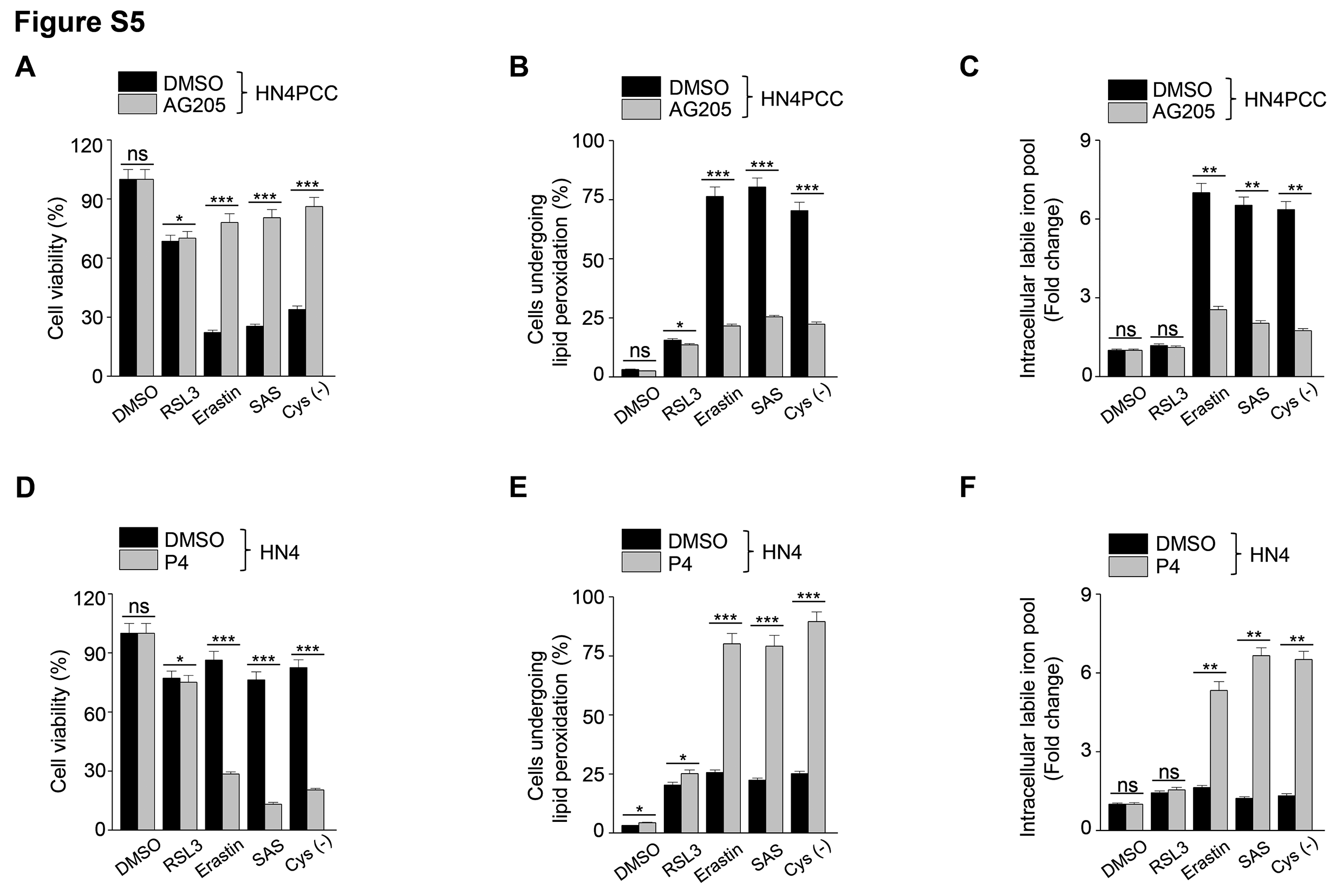


**Figure S5.** PGRMC1 expression is related to ferroptosis sensitivity. (***A***–***C***) Cell viability, lipid peroxidation, and labile iron pool (LIP) in HN4PCC with or without pharmacological inhibition of PGRMC1. The cells were treated with DMSO or 20 µM AG205, a PGRMC1 antagonist, and co-treated with 1 µM RSL3, 10 µM erastin, 0.5 mM SAS, and cyst(e)ine deprivation. Cell viability was examined using CCK-8 assay after 48 h treatment. Lipid peroxidation was examined using BODIPY^TM^ C11 and FACS after 8 h treatment. Intracellular LIP was measured using 8 μg/ml calcein-AM after 8 h treatment and quantified by ImageJ. Data are means and s.d. from three technical replicates. ns, non-significance; * *P* < 0.05, ***P* < 0.01, ****P* < 0.001 relative to DMSO control. (***D***–***F***) Cell viability, lipid peroxidation, and LIP in HN4 parental cells with or without progesterone (P4). The cells were treated with DMSO or 100 nM P4 and co-treated with the ferroptosis inducers.


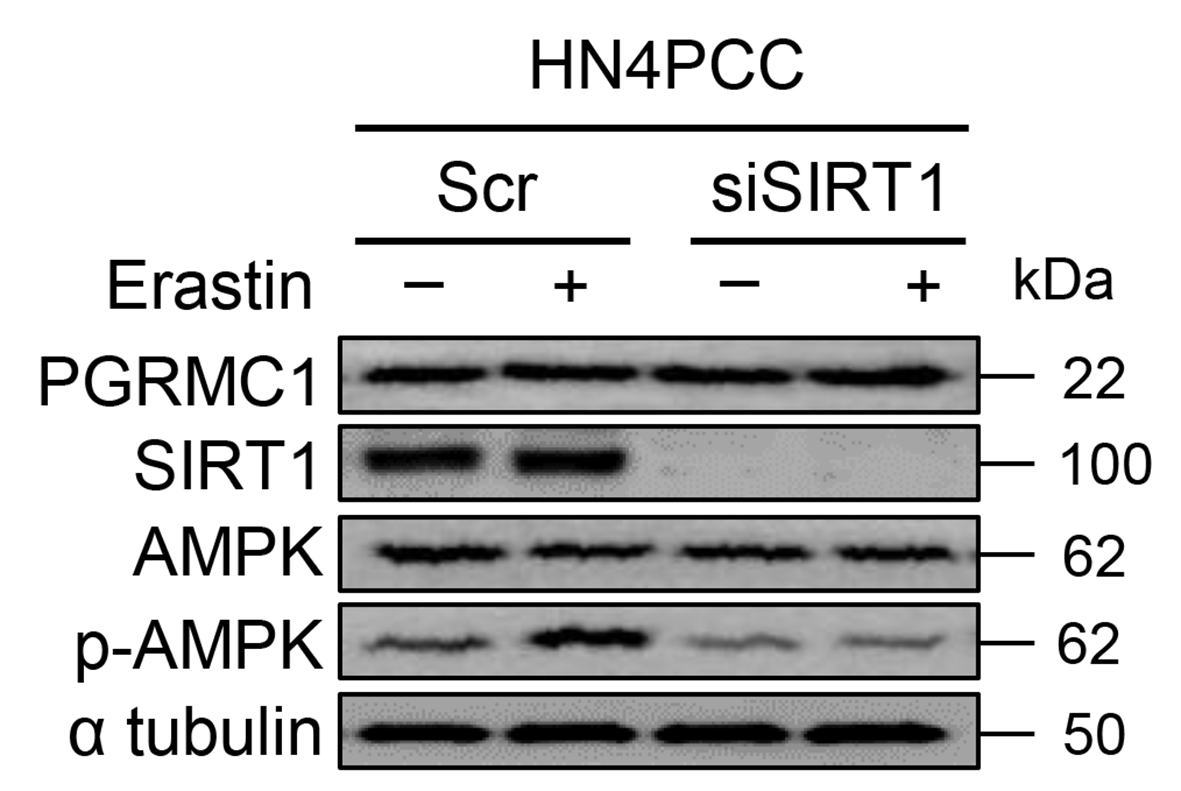


**Figure S6.** Immunoblotting in HN4 PCC with scrambled or SIRT1 siRNA transfection and then treated with DMSO or 10 µM erastin for 24 h.

**Table S1.** Correlation of mRNA expression levels between PGRMC1 and other genes from the HNC datasets of TCGA

|  | | PGRMC1 | |
| --- | --- | --- | --- |
| Gene | *r* ^a^ | | *P* |
| CD36 | 0.057 | | 0.183 |
| PNPLA2 | 0.013 | | 0.761 |
| SQSTM1 | 0.290 | | 5.108×10^-12^ |
| ATG5 | 0.328 | | 3.557×10^-15^ |
| MAP1LC3A | 0.031 | | 0.464 |
| MAP1LC3B | 0.084 | | 0.049 |
| SIRT1 | 0.357 | | 6.944×10^-18^ |
| ^a^ Pearson correlation coefficient (*r*), *P* < 0.05. | | | |
